# Interactions between dietary acrylamide intake and genes for ovarian cancer risk

**DOI:** 10.1007/s10654-017-0244-0

**Published:** 2017-04-08

**Authors:** Janneke G. F. Hogervorst, Piet A. van den Brandt, Roger W. L. Godschalk, Frederik-Jan van Schooten, Leo J. Schouten

**Affiliations:** 1 Centre for Environmental Sciences, Hasselt University, Diepenbeek, Belgium; 2 Department of Epidemiology, School for Oncology and Developmental Biology (GROW), Maastricht University, Maastricht, The Netherlands; 3 Department of Pharmacology and Toxicology, School for Nutrition and Translational Research in Metabolism (NUTRIM), Maastricht University, Maastricht, The Netherlands

**Keywords:** Dietary acrylamide, Single nucleotide polymorphism, Ovarian cancer, Prospective cohort

## Abstract

Some epidemiological studies observed a positive association between dietary acrylamide intake and ovarian cancer risk but the causality needs to be substantiated. By analyzing gene-acrylamide interactions for ovarian cancer risk for the first time, we aimed to contribute to this. The prospective Netherlands Cohort Study on diet and cancer includes 62,573 women, aged 55–69 years. At baseline in 1986, a random subcohort of 2589 women was sampled from the total cohort for a case cohort analysis approach. Dietary acrylamide intake of subcohort members and ovarian cancer cases (n = 252, based on 20.3 years of follow-up) was assessed with a food frequency questionnaire. We selected single nucleotide polymorphisms (SNPs) in genes in acrylamide metabolism and in genes involved in the possible mechanisms of acrylamide-induced carcinogenesis (effects on sex steroid systems, oxidative stress and DNA damage). Genotyping was done on DNA from toenails through Agena’s MassARRAY iPLEX platform. Multiplicative interaction between acrylamide intake and SNPs was assessed with Cox proportional hazards analysis. Among the results for 57 SNPs and 2 gene deletions, there were no statistically significant interactions between acrylamide and gene variants after adjustment for multiple testing. However, there were several nominally statistically significant interactions between acrylamide intake and SNPs in the *HSD3B1/B2* gene cluster: (rs4659175 (*p* interaction = 0.04), rs10923823 (*p* interaction = 0.06) and its proxy rs7546652 (*p* interaction = 0.05), rs1047303 (*p* interaction = 0.005), and rs6428830 (*p* interaction = 0.05). Although in need of confirmation, results of this study suggest that acrylamide may cause ovarian cancer through effects on sex hormones.

**Electronic supplementary material:**

The online version of this article (doi:10.1007/s10654-017-0244-0) contains supplementary material, which is available to authorized users.

## Introduction

Acrylamide, a probable human carcinogen (IARC class 2A; based on rodent studies), was discovered in 2002 in various heat-treated carbohydrate-rich foods, such as cookies, potato chips, French fries and coffee. Since then, epidemiological studies have been performed in order to investigate the impact of dietary acrylamide intake on human cancer risks. The results of these studies are inconsistent: for some cancers (endometrial, ovarian, breast and kidney cancer) increased risks have been observed in some studies but not all [1]. The outcome of a recent meta-analysis was that acrylamide intake was positively associated with an increased risk of ovarian cancer among never-smoking women (hazard ratio for high versus low intake: 1.39, 95% CI: 0.97–2.00) [1]. On the other hand, a recent study from the EPIC cohort published after the meta-analysis did not show an association [2] as did two studies using acrylamide biomarkers to estimate dietary acrylamide exposure instead of food frequency questionnaires [3, 4].

In the most recent risk assessment of acrylamide by the European Food Safety Authority (EFSA) [5], the epidemiological findings on acrylamide and cancer risk are discussed but not incorporated in the actual risk assessment. The most important reasons are the inconsistency in the findings and the fact that the causality of the observed associations between acrylamide intake and cancer risk is unclear. However, the risks observed in humans are considerably higher than predicted from rodent studies [6] and therefore we need to urgently get more clarity on the association between acrylamide intake and ovarian cancer risk and its causality.

In the present study, we aimed to investigate whether genetic make-up modifies the association between acrylamide and ovarian cancer risk, thereby contributing to evidence on acrylamide’s mechanism of action and the causality of the observed association in humans. Identification of stronger associations between acrylamide and ovarian cancer in genetically susceptible individuals (e.g., of a certain *CYP2E1* genotype) increases confidence that the observed association between acrylamide intake and ovarian cancer is not due to chance or bias. In addition, choosing genes that are relevant to the biological pathways of the disease can help to tease out disease-causing mechanisms of acrylamide. Finally, acrylamide is part of a mixture of heat-generated compounds or unhealthy diet which impairs the interpretation of acrylamide being the causative agent. Focusing on genes that are rather specific to acrylamide metabolism (e.g., *CYP2E1*) facilitates this interpretation.

We selected SNPs in candidate genes involved in acrylamide metabolism and in mechanisms through which acrylamide is hypothesized to cause cancer: mechanisms involving sex hormones, oxidative stress, and DNA damage caused by glycidamide, acrylamide’s genotoxic metabolite [7]. Previously, we investigated the interaction between genetic make-up and acrylamide intake for endometrial cancer risk, and we observed indications for interaction with SNPs in *CYP2E1* and the deletions of *GSTM1* and *GSTT1* [8].

## Subjects and methods

### Study cohort, cases and follow-up

The Netherlands Cohort Study on diet and cancer started in September 1986 with the inclusion of 62,573 women, 55–69 years of age. Data on dietary habits and other risk factors were collected by means of a self-administered questionnaire at baseline in 1986. Approximately 75% of the participants sent in toenail clippings, as requested.

Following the case-cohort approach, ovarian cancer cases, detected by annual computerized record linkages to the Netherlands Cancer Registry and the Netherlands Pathology Registry, were enumerated for the entire cohort, while the accumulated person-years for the entire cohort were estimated from a subcohort of 2589 women randomly sampled from the entire cohort at baseline. This study was approved by the review boards of TNO Nutrition and Food Research (Zeist, the Netherlands) and Maastricht University (Maastricht, the Netherlands). Written informed consent was provided by participants by returning the completed questionnaire. Further details on the design and methods of the study are presented elsewhere [9–12].

**Fig. 1:**
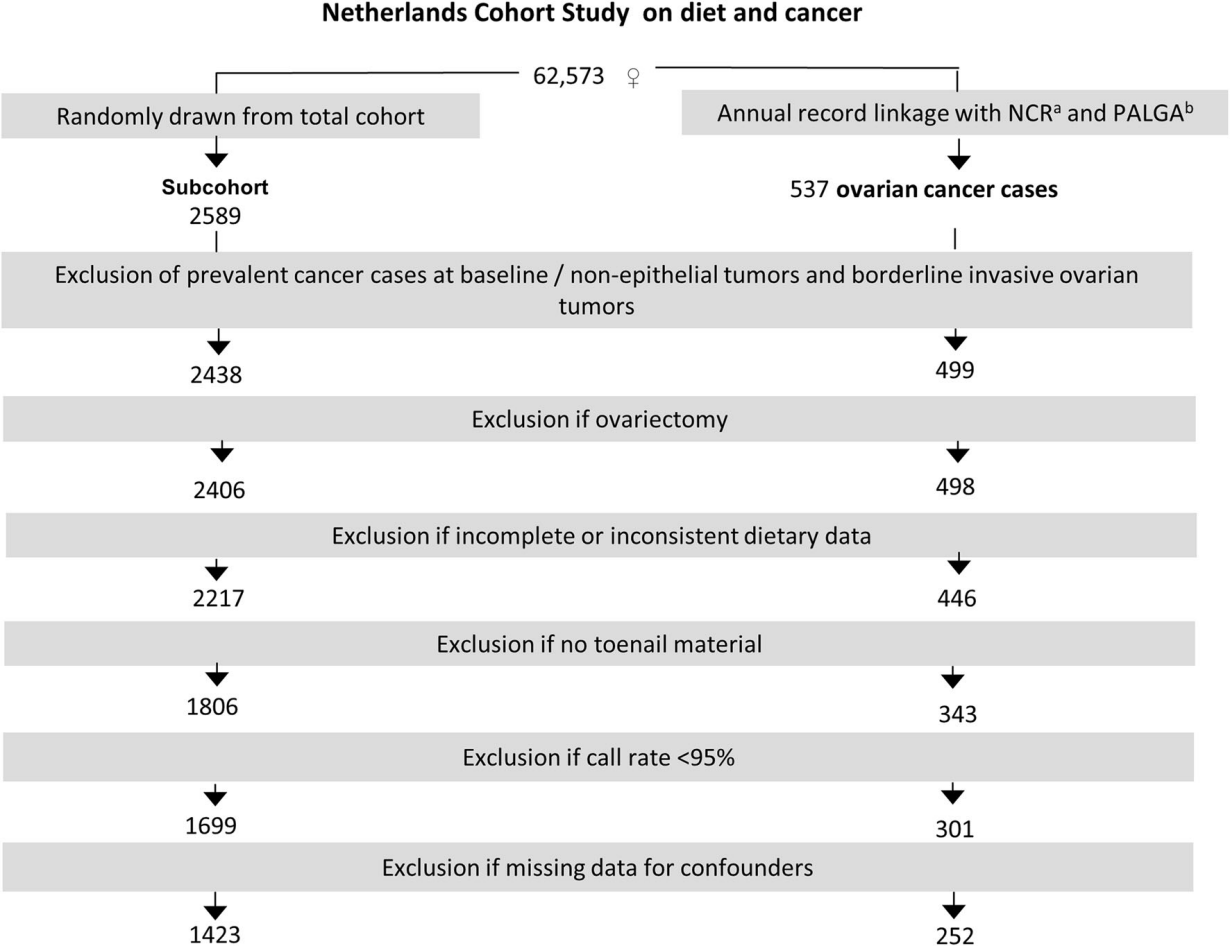
Flow chart of exclusion steps for ovarian cancer cases and subcohort members

After 20.3 years of follow-up, Sept. 1986–Dec. 2006, there were 499 microscopically confirmed invasive primary carcinomas of the ovaries ([ICD-O]-3: C56.9). Cases and subcohort members were excluded from analysis if they reported a diagnosis of cancer (except skin cancer) at baseline, their dietary data were incomplete or inconsistent, if they had not sent in toenail clippings, if they had no or inferior (call rate <95%) data on SNPs or if they reported at baseline to have had a unilateral or bilateral ovariectomy (see Fig. 1).

### Acrylamide intake assessment

A valid and reproducible food frequency questionnaire with questions on 150 food items was used for estimating dietary habits [11, 12]. Dietary acrylamide intake was estimated from the mean acrylamide level of foods on the Dutch market, and the frequency of consumption and portion size of the foods, as described in detail elsewhere [13].

### Selection of genes and SNPs

The selection of genes was broad and focused on genes involved in (1) acrylamide metabolism and (2) the most often hypothesized mechanisms of acrylamide-induced carcinogenesis [7]: (2a) sex hormonal effect (involving sex hormone synthesis/metabolism or sex hormone nuclear receptors), (2b) oxidative stress and (2c) genotoxicity (DNA repair), or (2d) SNPs in genes that otherwise clearly play a role in carcinogenesis. Genes and SNPs of interest were identified from the literature (HugeNavigator and PubMed) and from a personal communication (for SNP rs1280350 in *MGC12965*) with Jos Kleinjans (Dept. of Toxicogenomics, Maastricht University). Genes from category 2a (sex hormonal pathway) were selected based on the KEGG pathway Steroid Hormone Biosynthesis (map00140). Further details on the selection of genes and SNPS were reported elsewhere [8].

In the end, we genotyped 6 SNPs to determine the *GST* deletions and 60 SNPs in other genes, see Supplemental Table 1.

### DNA isolation and genotyping

DNA was isolated from 15 mg of toenail clippings, following the protocol developed by Cline et al. [14], in an optimised form [15]. Genotyping was performed by Agena in Hamburg, on the MassARRAY platform using the iPLEX TM assay [16]. This method has been used before to successfully genotype DNA from toenails [8, 15, 17, 18].

Supplemental Table 2 shows the 60 SNPs with their location, call frequencies, and HWE *p* value. 3 out of the 60 SNPs had a call rate <80% and were not included in the analyses. 6 SNPs out of the remaining 57 SNPs did not adhere to Hardy–Weinberg equilibrium (HWE) (p < 0.05). With regard to the SNPs selected to represent the *GSTM1* deletion, rs10857795 was not called in 36%, rs200184852 in 42% and rs74837985 in only 2% of the subcohort. The latter value appears to be due to genotyping error. Therefore, we decided to base the assessment of the absence/presence of the *GSTM1* gene only on rs10857795 and rs200184852. 31% of the subcohort had a missing value for both rs10857795 and rs200184852. With regard to *GSTT1*, rs2844008 was not called in 58%, rs4630 in 16%, and rs140309 in 11% of the subcohort. 8% of the subcohort had a missing value for all 3 *GSTT1* SNPs.

5% of the samples (n = 190) were duplicate samples to check the reproducibility of genotyping, which was >99%. We excluded samples with a call rate <95% (42 ovarian cancer cases, 107 subcohort members).

### Statistical analysis

Hazard rate ratios (HRs) and 95% confidence intervals were obtained through Cox proportional hazards regression with STATA software (package 13), using the robust Huber–White sandwich estimator to account for additional variance introduced by sampling from the cohort. The proportional hazards assumption was tested using scaled Schoenfeld residuals.

Acrylamide was included in the statistical models as a continuous variable and as quintiles for the main effect of acrylamide and as tertiles in the acrylamide-SNP interaction analyses.

Covariables were selected based on the literature: age, body mass index, height, age at menarche, age at menopause, use of oral contraceptives, parity, use of postmenopausal hormones, and energy intake. Smoking status, the duration of smoking and the number of cigarettes per day were included in the model, because cigarette smoke contains acrylamide[16, 17]. Furthermore, subgroup analyses were performed for never-smokers.

Multiplicative interaction between acrylamide intake and SNPs was tested using product terms of the continuous acrylamide intake variable and genotype. For statistical power reasons, we used a dominant genetic model for all SNPs (i.e., 1 or 2 variant alleles versus homozygous wild type). Tests for acrylamide dose–response trends in genotype strata were performed by fitting the mean acrylamide intake in the tertiles as a continuous variable.

We applied the False Discovery Rate method by Benjamini–Hochberg [19] to adjust for multiple testing with the expected proportion of false positives set at 20%, which is applied regularly in candidate gene studies [20, 21]. We performed separate adjustment for multiple testing for all women and for never-smoking women.

Two-sided *p* values are reported throughout.

## Results

Table 1 shows the characteristics of the participants at baseline. Cases were more often never-smokers, and had smoked less and for a shorter duration than subcohort members. They had less often used oral contraceptives. In addition, cases had fewer children.

**Table 1:** characteristics of subcohort and ovarian cancer cases

Variable	Ovarian cancer cases	Subcohort
n^a^	364	1474
*Dietary variables*		
Acrylamide intake (μg/day)	21.9 (13.1)	20.9 (11.8)
Total energy intake (kcal)	1684 (400)	1689 (399)
*Non*-*dietary variables*		
Age (yrs)	61.4 (4.3)	61.4 (4.3)
Body mass index (kg/m^2^)	25.0 (3.6)	25.1 (3.6)
Age at menarche (yrs)	13.7 (1.8)	13.7 (1.8)
Age at menopause (yrs)	49.0 (4.1)	48.8 (4.4)
Parity, n children	2.4 (2.2)	2.8 (2.2)
n cigarettes per day	3.5 (6.9)	4.5 (7.7)
n smoking years	9.1 (14.5)	11.3 (15.7)
*Cigarette smoking status* %		
Never smokers	64.8	58.7
Former smokers	19.6	20.9
Current smokers	15.6	20.4
Ever use of postmenopausal hormone treatment, % yes	12.1	13.3
Ever use of oral contraceptives, % yes	16.4	25.4

^a^n represents number of subcohort members or cases after exclusion of participants with prevalent cancer at baseline, ovariectomy, incomplete or inconsistent dietary data, and a sample call rate <95%. The number of missing values varies for the variables in this Table

### Main effect of acrylamide

There was a suggestive (statistically non-significant) positive association between acrylamide and ovarian cancer risk after 20.3 years of follow-up (HR of highest versus the lowest quintile of intake: 1.38 (95% CI 0.95–1.99) and 1.06 (0.98–1.16) per 10 µg/day increment of intake), which was stronger and statistically significant among never-smoking women (HR of highest versus the lowest quintile of intake: 1.85 (95% CI 1.15–2.95) and 1.15 (1.02–1.30) per 10 µg/day increment of intake) (Table 2).

**Table 2:** Main association between acrylamide intake and ovarian cancer risk, 20.3 years of follow-up

	n cases	Per 10 µg/day increment	Quintile 1	Quintile 2	Quintile 3	Quintile 4	Quintile 5	*p* trend
		HR (95% CI)^a^	HR (95% CI)	HR (95% CI)	HR (95% CI)	HR (95% CI)	HR (95% CI)	
All women	373	1.06 (0.98–1.16)	Ref (1.00)	1.07 (0.73–1.54)	1.10 (0.75–1.61)	1.05 (0.71–1.53)	1.38 (0.95–1.99)	0.13
Never-smoking women	243	1.15 (1.02–1.30)	Ref (1.00)	1.37 (0.85–2.21)	1.61 (0.98–2.65)	1.50 (0.92–2.44)	1.85 (1.15–2.95)	0.01

Hazard ratios are adjusted for age (years), age at menarche (years), age at menopause (years), parity (n children), ever use of oral contraceptives (yes/no), ever use of postmenopausal hormone treatment (yes/no), height (cm), body mass index (kg/m^2^), energy intake (kcal/day), and in the analyses for all women: smoking status (never/ex/current smoker), smoking quantity (n cigarettes/day), smoking duration (smoking years)

The median acrylamide intake of the female subcohort in the quintiles was 9.5, 14.0, 17.9, 24.3, and 36.8 μg/day

^a^HR (95% CI): hazard ratio with corresponding 95% confidence interval

### Main effect of the SNPs

Table 3 presents the SNPs showing a clear trend for ovarian cancer over the number of variant alleles. There was an increase in risk with an increasing number of variant alleles for rs511895 in *CAT* (*p* trend = 0.04), rs1056827 in *CYP1B1* (*p* trend = 0.06), and rs2301241 in *TXN* (*p* trend = 0.02). Decreased risks were observed for rs4646903 in *CYP1A1* (*p* = 0.06), rs3219489 in *MUTYH* (*p* trend = 0.05) and the homozygous deletion of *GSTM1* (*p* = 0.03). However, none of the SNPs was statistically significantly associated with ovarian cancer risk after adjustment for multiple comparisons.

**Table 3:** Genetic variants showing a clear dose–response relationship in their association with ovarian cancer risk, 20.3 years of follow-up

Main effects SNPs	Homozygous wildtype		1 or 2 variant alleles		1 variant allele		2 variant alleles		*p* trend per allele	Benjamini–Hochberg-adjusted *p* value
	N cases	HR (95% CI)^a^	N cases	HR (95% CI)^a^	N cases	HR (95% CI)^a^	N cases	HR (95% CI)^a^		
*CAT*, rs511895	86	Ref	215	1.25 (0.95–1.63)	154	1.17 (0.88–1.56)	61	1.48 (1.04–2.13)	0.04	0.59
*CYP1A1*, rs4646903	261	Ref	36	0.70 (0.48–1.02)	36	0.70 (0.48–1.02)		na	0.06	0.59
*CYP1B1*, rs1056827	144	Ref	154	1.26 (0.99–1.62)	127	1.24 (0.96–1.61)	27	1.36 (0.87–2.14)	0.06	0.59
*MUTYH*, rs3219489	189	Ref	112	0.78 (0.60–1.00)	97	0.79 (0.61–1.03)	15	0.70 (0.40–1.23)	0.05	0.59
*TXN*, rs2301241	95	Ref	206	1.26 (0.97–1.65)	147	1.18 (0.89–1.56)	59	1.55 (1.08–2.22)	0.02	0.59

*GSTM1* deletion	1 or 2 alleles present		Homozygous deletion		*p* value	Benjamini–Hochberg-adjusted *p* value
	N cases	HR (95% CI)^a^	N cases	HR (95% CI)^a^		
Deletion represented by						
Both *GSTM1* SNPs	226	Ref	75	0.74 (0.56–0.98)	0.03	0.59
rs10857795	214	Ref	87	0.73 (0.56–0.95)	0.02	0.59
rs200184852	185	Ref	116	0.84 (0.66–1.09)	0.19	0.59

^a^HR (95% CI): hazard ratio with corresponding 95% confidence interval; hazard ratios are adjusted for age; *na* not applicable

### Interaction between acrylamide and SNPs

None of the SNPs showed a statistically significant multiplicative interaction with acrylamide after adjustment for multiple comparisons. In Table 4, we show interactions with SNPs in genes involved in acrylamide metabolism that are interesting because they have a higher *a priori* probability of modifying the association between acrylamide and cancer risk than the other selected SNPs. Rs915906 and rs2480258 in *CYP2E1* did not show a statistically significant interaction with acrylamide intake among all women (*p* interaction = 0.52 and 0.45, respectively) nor among never-smoking women (*p* interaction = 0.92 and 0.87, respectively). However, for both SNPs, acrylamide was only positively associated with ovarian cancer risk in women homozygous for the wild type allele and in never-smokers, there was a clear but statistically non-significant dose–response trend for acrylamide for rs915906 (*p* trend = 0.08) and a clear and statistically significant dose–response trend for rs2480258 (*p* trend = 0.04). The homozygous deletion of *GSTT1* did not show an interaction with acrylamide intake but when the deletion was represented by rs4630, acrylamide was only positively associated with ovarian cancer risk in women with at least 1 copy of the *GSTT1* gene, with a *p* for trend of 0.09 among all women and 0.05 among never-smokers. There was no interaction between the deletion of *GSTM1* or other SNPs in acrylamide-metabolizing genes and acrylamide, and no clear difference in the acrylamide-associated risk between the genotypes of these genes.

**Table 4:** Interactions between SNPs in acrylamide-metabolizing genes and dietary acrylamide intake on the risk of ovarian cancer, 20.3 years of follow-up

SNP^a^	Acrylamide, continuous intake	Acrylamide, tertiles of intake							Interaction	
	10 µg/day	Tertile 1		Tertile 2		Tertile 3		*p* for trend	*p* for linear interaction	
		N cases	HR (95% CI)^c^	N cases	HR (95% CI)^c^	N cases	HR (95% CI)^c^		Raw p	Benjamini–Hochberg adjusted p value
All										
*CYP2E1*, rs915906 = 0^b^	1.12 (0.99–1.26)	55	Ref (1.00)	50	0.98 (0.64–1.50)	78	1.35 (0.91–2.01)	0.12	0.52	0.81
*CYP2E1*, rs915906 = 1^b^	1.00 (0.76–1.32)	33	Ref (1.00)	14	0.42 (0.20–0.87)	22	0.65 (0.33–1.27)	0.21		
Never-smokers										
*CYP2E1*, rs915906 = 0	1.18 (1.01–1.38)	32	Ref (1.00)	38	1.36 (0.80–2.32)	49	1.57 (0.95–2.59)	0.08	0.92	0.96
*CYP2E1*, rs915906 = 1	1.09 (0.77–1.53)	20	Ref (1.00)	9	0.45 (0.17–1.19)	15	0.72 (0.30–1.72)	0.47		
All										
*CYP2E1*, rs2480258 = 0	1.13 (0.99–1.28)	51	Ref (1.00)	47	1.03 (0.66–1.62)	73	1.40 (0.93–2.13)	0.10	0.45	0.78
*CYP2E1*, rs2480258 = 1	0.98 (0.79–1.22)	37	Ref (1.00)	17	0.43 (0.22–0.84)	27	0.66 (0.37–1.20)	0.18		
Never-smokers										
*CYP2E1*, rs2480258 = 0	1.19 (1.02–1.40)	30	Ref (1.00)	36	1.52 (0.87–2.64)	47	1.75 (1.04–2.97)	0.04	0.87	0.96
*CYP2E1*, rs2480258 = 1	1.07 (0.78–1.48)	22	Ref (1.00)	11	0.43 (0.18–1.02)	17	0.59 (0.26–1.34)	0.24		
All										
*CYP2E1*, rs6413432 = 0	1.07 (0.96–1.19)	71	Ref (1.00)	60	0.94 (0.66–1.34)	85	1.09 (0.79–1.52)	0.58	0.88	0.93
*CYP2E1*, rs6413432 = 1	1.04 (0.74–1.47)	17	Ref (1.00)	4	0.19 (0.06–0.57)	15	0.76 (0.29–1.97)	0.49		
Never-smokers										
*CYP2E1*, rs6413432 = 0	1.09 (0.94–1.25)	46	Ref (1.00)	44	1.07 (0.70–1.65)	54	1.05 (0.69–1.58)	0.83	0.19	0.65
*CYP2E1*, rs6413432 = 1	1.49 (0.89–2.49)	6	Ref (1.00)	3	0.20 (0.04–1.06)	10	0.92 (0.24–3.49)	0.98		
All										
*GSTM1* present, all SNPs	1.07 (0.94–1.22)	65	Ref (1.00)	48	0.79 (0.51–1.21)	76	1.09 (0.73–1.61)	0.62	0.73	0.90
*GSTM1* deleted, all SNPs	1.15 (0.90–1.47)	23	Ref (1.00)	16	0.65 (0.31–1.35)	24	1.02 (0.50–2.08)	0.92		
Never-smokers										
*GSTM1* present, all SNPs	1.13 (0.96–1.32)	40	Ref (1.00)	34	1.04 (0.60–1.79)	47	1.25 (0.76–2.05)	0.37	0.43	0.76
*GSTM1* deleted, all SNPs	1.29 (0.89–1.86)	12	Ref (1.00)	13	1.07 (0.43–2.62)	17	1.25 (0.51–3.03)	0.62		
All										
*GSTT1* present, rs4630	1.15 (1.03–1.29)	68	Ref (1.00)	52	0.83 (0.56–1.25)	89	1.36 (0.94–1.97)	0.09	0.11	0.67
*GSTT1* deleted, rs4630	0.79 (0.53–1.19)	20	Ref (1.00)	12	0.50 (0.20–1.24)	11	0.31 (0.12–0.77)	0.01		
Never-smokers										
*GSTT1* present, rs4630	1.23 (1.06–1.44)	40	Ref (1.00)	41	1.14 (0.69–1.87)	57	1.59 (0.99–2.54)	0.05	0.26	0.65
*GSTT1* deleted, rs4630	0.87 (0.53–1.44)	12	Ref (1.00)	6	0.52 (0.15–1.81)	7	0.34 (0.10–1.22)	0.10		
All										
*GSTP1*, rs1695 = 0	1.05 (0.88–1.25)	31	Ref (1.00)	32	0.96 (0.58–1.58)	38	0.99 (0.59–1.66)	0.98	0.81	0.90
*GSTP1*, rs1695 = 1	1.07 (0.94–1.23)	57	Ref (1.00)	32	0.63 (0.41–0.97)	62	1.02 (0.70–1.50)	0.90		
Never-smokers										
*GSTP1*, rs1695 = 0	1.07 (0.85–1.36)	19	Ref (1.00)	25	1.17 (0.65–2.11)	24	0.91 (0.48–1.70)	0.74	0.79	0.96
*GSTP1*, rs1695 = 1	1.13 (0.95–1.34)	33	Ref (1.00)	22	0.73 (0.42–1.26)	40	1.09 (0.66–1.79)	0.74		
All										
*GSTA5*, rs4715354 = 0	0.98 (0.80–1.20)	24	Ref (1.00)	20	1.20 (0.56–2.54)	25	1.06 (0.53–2.13)	0.87	0.56	0.81
*GSTA5*, rs4715354 = 1	1.13 (0.99–1.28)	64	Ref (1.00)	44	0.71 (0.46–1.08)	75	1.15 (0.78–1.69)	0.43		
Never-smokers										
*GSTA5*, rs4715354 = 0	1.03 (0.80–1.32)	14	Ref (1.00)	13	1.48 (0.55–3.94)	19	1.33 (0.56–3.13)	0.55	0.61	0.83
*GSTA5*, rs4715354 = 1	1.21 (1.00–1.46)	38	Ref (1.00)	34	0.97 (0.58–1.65)	45	1.25 (0.75–2.07)	0.38		
All										
*EPHX1*, rs1051740 = 0	1.06 (0.89–1.27)	46	Ref (1.00)	26	0.55 (0.34–0.89)	47	0.86 (0.55–1.35)	0.55	0.87	0.93
*EPHX1*, rs1051740 = 1	1.07 (0.94–1.22)	42	Ref (1.00)	38	0.98 (0.62–1.53)	53	1.19 (0.78–1.81)	0.41		
Never-smokers										
*EPHX1*, rs1051740 = 0	1.10 (0.88–1.38)	31	Ref (1.00)	20	0.63 (0.36–1.12)	31	0.79 (0.46–1.37)	0.41	0.88	0.96
*EPHX1*, rs1051740 = 1	1.12 (0.94–1.33)	21	Ref (1.00)	27	1.32 (0.74–2.36)	33	1.36 (0.77–2.40)	0.30		

Hazard ratios are adjusted for age (years), age at menarche (years), age at menopause (years), parity (n children), ever use of oral contraceptives (yes/no), ever use of postmenopausal hormone treatment (yes/no), height (cm), body mass index (kg/m^2^), energy intake (kcal/day), and in the analyses for all women: smoking status (never/ex/current smoker), smoking quantity (n cigarettes/day), smoking duration (smoking years)

The median acrylamide intake of the female subcohort in the quintiles was 9.5, 14.0, 17.9, 24.3, and 36.8 μg/day

^a^SNP: single nucleotide polymorphism

^b^0: homozygous wildtypes, 1: 1 or 2 variant alleles

^c^HR (95% CI): hazard ratio with corresponding 95% confidence interval

Supplemental Table 3 shows the results for other SNPs that showed an interaction with acrylamide, or for which the acrylamide-associated risk of ovarian cancer clearly differed between the genotypes. For 5 SNPs in the *HSD3B1/B2* gene cluster, namely rs4659175 (*p* interaction = 0.04), rs10923823 (*p* interaction = 0.06) and its proxy rs7546652 (*p* interaction = 0.05), rs1047303 (*p* interaction = 0.005), and rs6428830 (*p* interaction = 0.05), the acrylamide dose–response relationships differed importantly between the genotypes. For all these SNPs, acrylamide intake was only clearly positively associated with ovarian cancer risk among women with 1 or 2 variant alleles. Among never-smoking women, the difference between the genotypes was more pronounced.

## Discussion

The current study is the first to analyze acrylamide-gene interactions for ovarian cancer risk. We carefully selected SNPs in genes involved in acrylamide metabolism and genes involved in pathways involved in the mechanism by which acrylamide might cause cancer: a sex hormonal effect, oxidative stress and DNA damage, or otherwise.

### CYP2E1

Glycidamide (formed by epoxidation of acrylamide through CYP2E1) is often thought to be the compound responsible for acrylamide-induced carcinogenesis due to genotoxicity. Therefore, studying the modifying effect of SNPs in *CYP2E1* on the association between acrylamide and cancer risk contributes important information on the causality of the association. There was no statistically significant interaction between the 3 studied SNPS in *CYP2E1* and acrylamide intake for ovarian cancer risk. However, similar to endometrial cancer risk [8], where nominally statistically significant interactions were observed for rs915906 and rs2480258, we observed increased acrylamide-associated risks of ovarian cancer only in women homozygous for the wild type allele of both SNPs. As discussed previously [8], this would suggest that acrylamide itself is the causative compound in ovarian carcinogenesis, because the strongest association between acrylamide and ovarian cancer risk was observed among homozygous wild types, suggesting another mechanism of action than genotoxicity. Rs2480258 in *CYP2E1* was not in Hardy–Weinberg equilibrium, although with a minor deviation (*p* = 0.03). This may indicate that the genotypes for this SNP were measured with some error but there is no reason to assume that this error is different for cases and subcohort members or for different categories of acrylamide intake. Therefore, this potential genotyping error would rather lead to missing a true interactions, if any [22].

### GSTs

We observed that women with at least one copy of *GSTT1* were at an increased acrylamide-associated risk of ovarian cancer, which was also what we observed for endometrial cancer [8] but the number of cases with a homozygous deletion of the *GSTT1* gene was very small (n = 43). Also similar to endometrial cancer, the homozygous deletion of *GSTM1* was nominally statistically significantly associated with a reduced risk of ovarian cancer, and the homozygous deletion of *GSTT1* was statistically non-significantly associated [among all women: HR: 0.59 (0.18–1.95); never-smokers: HR: 0.58 (0.13–2.55)] with a reduced risk of ovarian cancer. In a recent meta-analysis, there was no association between the null genotypes of *GSTM1* and *GSTT1* and ovarian cancer risk [23]. Unlike for endometrial cancer, there was no difference in the association between acrylamide intake and ovarian cancer risk between the genotypes of *GSTM1*.

A possible explanation for the inverse association between the null genotypes of *GSTM1* and *GSTT1* and ovarian cancer risk is that GSTs catalyze the conjugation of reduced glutathione (GSH) to compounds that protect against ovarian cancer or that they bioactivate compounds involved in ovarian carcinogenesis, for instance catechol estrogens [24]. Conjugation of acrylamide with GSH can result in depletion of cellular GSH stores, leading to altered gene expression directly or through regulating various redox-dependent transcription factors [7]. Considering the fact that acrylamide induces GST activity [25, 26], it would be expected that the positive association between acrylamide and ovarian cancer is only present among women with at least one copy of the genes in whom the activity of GST can be induced.

### Hsd3b1/2

We observed nominally statistically significant interaction between acrylamide intake and 5 SNPs in the *HSD3B1/B2* gene cluster of which 2 were complete proxies: rs7546652 and rs10923823 (R^2^ = 1, D’ = 1). The 3b-hydroxysteroid dehydrogenase/δ5-4 is a key rate-limiting enzyme in steroid biosynthesis pathways producing progesterone and androgens. Two studies in mice have shown that acrylamide down-regulated the expression of *HSD3B2.* (personal communication with Prof. Nan Mei, December 2014 + [25]) Acrylamide has repeatedly been shown to decrease progesterone and testosterone levels in mice and rats [27–29]. Thus, although speculative, the observed interactions between SNPs in the *HSD3B* genes and acrylamide suggest that acrylamide may be involved in ovarian carcinogenesis through effects on progesterone or androgens, since progesterone probably suppresses ovarian carcinogenesis [30–35], and androgens may induce ovarian carcinogenesis [35]. A cross-sectional study on the association between acrylamide intake and progesterone in premenopausal women found no indications for an association between the two but in the same study there were positive associations between acrylamide intake and DHEAS and testosterone in overweight postmenopausal women [36].

### Other genes

In addition, for some SNPs, there were no statistically significant indications for interaction but still a clear difference (strongest among never-smokers) in the association between acrylamide intake and ovarian cancer risk between the genotypes: rs11252859 in *AKR1C1* (also involved in progesterone and androgen metabolism), rs3448 in *GPX1*, rs11632903 in *CYP19A1*, rs1800566 in *NQO1*, rs1052133 in *OGG1*, rs824811 and rs8192120 in *SRD5A1* (also involved in progesterone and androgen metabolism), and rs2228000 in *XPC*, rs1056827 in *CYP1B1*, rs2987983 in *ESR2*, rs1280350 in *MGC12965*, rs944722 in *NOS2*, and rs5275 in *PTGS2.* It is, however, premature to elaborately discuss their possible role in acrylamide-induced ovarian carcinogenesis here.

Interactions between SNPs and acrylamide intake for both endometrial [8] and ovarian cancer (this paper) lacked statistical significance after adjustment for multiple testing, probably partly due to a lack of statistical power because in many instances there was a clear difference in the acrylamide-associated risk between genotypes. However, it is worthwhile to look at the overlap between the SNPs for both cancers. The following SNPs showed a nominally statistically significant interaction with acrylamide intake for both endometrial and ovarian cancer, with the same genotypes showing the strongest positive association between acrylamide and cancer risk in never-smokers: rs11252859 in *AKR1C1*, rs3448 in *GPX1*, and rs1800566 in *NQO1*. Additionally, there were clear differences in the acrylamide dose–response between the same genotypes for both cancers for: rs1280350 in *MGC1295* (among never-smokers), and rs6428830 in the *HSD3B1/B2* gene cluster (particularly among never-smokers). These SNPs are worthwhile investigating in future studies on acrylamide intake and endometrial and ovarian cancer risk.

### Limitations

This study has some limitations. In the present analysis for ovarian cancer, acrylamide intake was statistically significantly associated with an increased ovarian cancer risk after 20.3 years of follow-up, while the association was only present in the first 11.3 years of follow-up for endometrial cancer [8]. We have no clear explanation for this but it is possible that, due to the fact that endometrial and ovarian cancer are different tumors with a different etiology and partly differing risk factors, acrylamide may have a different role in the etiology of these tumors. An example of the different etiologies of these cancers is that estrogens are thought to play a major role in the etiology of endometrial cancer [37], while they seem to less do so in the etiology of ovarian cancer, which seems to be more clearly influenced by progesterone and androgens [38].

Some of the interactions that we discussed are probably chance findings, considering that none of the SNPs survived adjustment for multiple comparisons. However, finding interactions for multiple SNPs in the *HSD3B1/B2* gene cluster decreases the likelihood that they are chance findings, especially with clear differences in the dose–response pattern of acrylamide between the genotypes.

The statistical power to detect interactions was probably too low for analyses where subgroups based on genotype and acrylamide intake category were small, especially when adjusted for multiple comparisons.

We were unable to assess dietary acrylamide intake with the acrylamide to hemoglobin adduct biomarker because we did not collect blood from the study participants. However, we are not convinced that using biomarkers to estimate acrylamide intake is always necessarily superior to using questionnaires. There are various reasons why acrylamide and glycidamide to hemoglobin adducts (AA and GA Hb-adducts) may not be perfect long-term exposure markers. AA and GA Hb-adducts display large intra-individual variability, as shown by Vikstrom et al. [39], which is probably due to variations in intake of acrylamide-containing foods. This is probably due to intermittent high intakes of foods containing high concentrations of acrylamide which considerably impact the value of the AA and GA Hb-adducts. Similar levels of adducts can arise from a low exposure over an extended time period and from a high incidental exposure. This is not desirable, because for investigating the relationship with cancer, it is probably more important to know the long-term average. Further, acrylamide and glycidamide Hb-adducts are expressed per gram of globin, which means that two persons with the same acrylamide intake may have different AA and GA Hb-adduct levels, dependent on their hemoglobin status. There are many factors that influence hemoglobin levels, such as sex, age, smoking, alcohol intake, physical exercise, and diet. In addition, the biomarker is not specific for the source of exposure and both active and passive smoking influence AA and GA Hb-adduct levels.

Strengths of this study are the complete follow-up, the prospective nature, and the fact that we observed a main association between acrylamide intake and endometrial and ovarian cancer risk, indicating that acrylamide intake was probably assessed reasonably well in this study.

## Conclusion

This study showed nominally statistically significant interactions between several SNPs in the *HSD3B1/B2* gene cluster and acrylamide intake for ovarian cancer risk, suggesting that acrylamide may cause ovarian cancer through effects on sex hormones. Based on this study and our study on endometrial cancer [8], we recommend follow-up of interactions between acrylamide intake and SNPs for ovarian and endometrial cancer risk, particularly SNPs in *CYP2E1*, *GSTs*, the *HSD3B1/B2* gene cluster, *AKR1C1*, *NQO1*, *GPX1* and *MGC12965*.

## Electronic supplementary material

Below is the link to the electronic supplementary material.

Supplementary material 1 (DOCX 435 kb)
