# Supplementary material for: Interactions between dietary acrylamide intake and genes for ovarian cancer risk
Source: Eur J Epidemiol. 2017 Apr 8;32(5):431–41. doi: 10.1007/s10654-017-0244-0 (PMC5506210; doi:10.1007/s10654-017-0244-0)
Supplement: Supplementary file 1 — Supplementary material 1 (DOCX 435 kb) [file 10654_2017_244_MOESM1_ESM.docx]

Supplemental Tables

Janneke GF Hogervorst, Piet A van den Brandt, Roger WL Godschalk, Frederik-Jan van Schooten, Leo J Schouten.^.^

Interactions between dietary acrylamide intake and genes for ovarian cancer risk.

Supplemental Table 1: Potentially interesting SNPs for investigating acrylamide-gene interactions (* eventually genotyped)

| **Gene** | **Reason for (non)selection (apart from association with cancer)** | | **db SNP ID** | **MAF** | **Localization in gene and predicted functionality** | **Association with cancer** |
| --- | --- | --- | --- | --- | --- | --- |
| ***Category 1: Acrylamide metabolism*** | | | | | | |
| ***CYP2E1*** | Polymorphism study[^1^](#_ENREF_1) | |  |  |  |  |
|  |  | |  |  |  |  |
|  |  | | rs2480258* | 25 | Intronic, no info in F-SNP | No leads in HugeNavigator and PubMed (in Dec 2013) |
|  |  | | rs915906* | 17 | Intronic, no info in F-SNP | No leads in HugeNavigator and PubMed (in Dec 2013) |
|  | *Not selected: MAF too low* | | rs2031920 | 6 | Regulatory region, upstream, 0.40 | Associated with prostate cancer risk[^2^](#_ENREF_2) and breast cancer risk[^3^](#_ENREF_3) |
|  |  | | rs6413432* | 14 | Intronic, no info in F-SNP | Associated with prostate cancer risk[^4^](#_ENREF_4) |
|  | *Not selected: MAF too low* | | rs3813867 | 7 | Upstream, no info in F-SNP |  |
| ***EPHX1*** | Gene expression study[^5^](#_ENREF_5)  + polymorphism studies[^1^](#_ENREF_1)^,^ [^6^](#_ENREF_6) | |  |  |  |  |
|  | Polymorphism study[^6^](#_ENREF_6) | | rs1051740* | 33 | NS coding, 0.70 | Associated with ovarian cancer risk[^7^](#_ENREF_7)^,^ [^8^](#_ENREF_8) + breast cancer risk[^9^](#_ENREF_9) + prostate cancer risk[^10^](#_ENREF_10) |
|  | Polymorphism studies[^1^](#_ENREF_1)^,^[^6^](#_ENREF_6) | | rs2234922 | 21 | NS coding, 0.14 | No leads in HugeNavigator and PubMed (in Dec 2013) |
| ***GSTA2*** | Polymorphism study [^11^](#_ENREF_11)  + gene expression study[^12^](#_ENREF_12) | |  |  |  |  |
|  | *Not selected: MAF too low* | | rs6577 | 7 | NS coding, 0.90 | No leads in HugeNavigator and PubMed (in Dec 2013) |
| ***GSTA5*** | Gene expression study[^13^](#_ENREF_13) | |  |  |  |  |
|  | Highest F-value, sufficient MAF | | rs4715354* | 49 | Intronic, 0.5 | No leads in HugeNavigator and PubMed(in Dec 2013) |
| ***GSTM1*** | Polymorphism studies[^1^](#_ENREF_1)^,^ [^6^](#_ENREF_6)^,^ [^14^](#_ENREF_14)  + gene expression study[^15^](#_ENREF_15) | |  |  |  |  |
|  | Polymorphism studies[^1^](#_ENREF_1)^,^ [^14^](#_ENREF_14) | | Gene deletion | 50 |  | Associated with breast cancer risk[^16^](#_ENREF_16) + prostate cancer risk[^17^](#_ENREF_17) + ovarian cancer risk[^7^](#_ENREF_7)^,^ [^18^](#_ENREF_18)^,^ [^19^](#_ENREF_19) + endometrial cancer risk[^20^](#_ENREF_20) |
|  | Selected to represent gene deletion | | rs200184852* | 22 | NS coding, no info in F-SNP | No leads in HugeNavigator and PubMed(in Dec 2013) |
|  | Selected to represent gene deletion | | rs74837985* | 42 | NS coding, no info in F-SNP | No leads in HugeNavigator and PubMed (in Dec 2013) |
|  | Selected to represent gene deletion | | rs1056806* | 13 | S coding, 0.27 | No leads in HugeNavigator and PubMed(in Dec 2013) |
| ***GSTM4*** | Gene expression study[^13^](#_ENREF_13) | |  |  |  |  |
|  |  | | rs560018 | 37 | Intronic, no info in F-SNP | No leads in HugeNavigator and PubMed(in Dec 2013) |
| ***GSTP1*** | Polymorphism study[^1^](#_ENREF_1)  + gene expression study[^21^](#_ENREF_21) | |  |  |  |  |
|  | Polymorphism study [^11^](#_ENREF_11) | | rs1695* | 39 | NS coding, 0.37 | Associated with breast cancer risk[^22^](#_ENREF_22)+ prostate cancer risk[^23^](#_ENREF_23) + endometrial cancer risk[^24^](#_ENREF_24) + ovarian cancer[^19^](#_ENREF_19) |
| ***GSTT1*** | Polymorphism studies[^1^](#_ENREF_1)^,^ [^14^](#_ENREF_14) | |  |  |  |  |
|  | Polymorphism studies[^1^](#_ENREF_1)^,^ [^14^](#_ENREF_14) | | Gene deletion | 18 |  | Associated with prostate cancer risk[^25^](#_ENREF_25)^,^ [^26^](#_ENREF_26) + breast cancer risk[^27^](#_ENREF_27) + endometrial cancer risk[^28^](#_ENREF_28) |
|  | Selected to represent gene deletion | | rs4630* | 11 | 3’-UTR, 0 | No leads in HugeNavigator and PubMed (in Dec 2013) |
|  | Selected to represent gene deletion | | rs140309* | 11 | 3’-UTR, 0.5 | No leads in HugeNavigator and PubMed (in Dec 2013) |
|  | Selected to represent gene deletion | | rs2844008* | 11 | Intron, 0.24 | No leads in HugeNavigator and PubMed (in Dec 2013) |
| ***Category 2a: Sex hormone metabolism and sex hormone nuclear receptors*** | | | | | | |
| ***AKR1C1*** | | Gene expression study[^5^](#_ENREF_5) |  |  |  |  |
|  | |  | rs11252859* | 38 | Downstream, 0.66 | No leads in HugeNavigator and PubMed (in Dec 2013) |
| ***AKR1C2*** | | Gene expression study[^5^](#_ENREF_5) |  |  |  |  |
|  | |  | rs11252887* | 32 | Intergenic, 0.5 | Associated with endometrial cancer risk[^29^](#_ENREF_29) |
| ***AKR1C3*** | | Gene expression study[^5^](#_ENREF_5) |  |  |  |  |
|  | |  | rs12529 | 40 | NS coding, 0.5 | Associated with prostate cancer prognosis[^30^](#_ENREF_30) |
|  | |  | rs7741* | 35 | S coding, conserved | Associated with hereditary prostate cancer risk[^26^](#_ENREF_26) |
|  | |  | rs12387 | 16 | S coding | Associated with breast cancer risk among PHT users[^31^](#_ENREF_31) |
|  | |  | rs4881400 | 24 | Intronic, no info in F-SNP | Associated with hereditary prostate cancer risk[^26^](#_ENREF_26)^,^ [^32^](#_ENREF_32) |
| ***COMT*** | | KEGG Pathway Steroid Hormone Biosynthesis |  |  |  |  |
|  | |  | rs4680* | 48 | NS coding, 0.86 | Associated with breast cancer risk meta-analysis[^33^](#_ENREF_33) + endometrial cancer risk[^34^](#_ENREF_34) |
|  | |  | rs737865 | 32 | Intronic, 0.18 | Associated with breast cancer risk[^35^](#_ENREF_35) |
| ***CYP1A1*** | | Gene expression study[^5^](#_ENREF_5) |  |  |  |  |
|  | |  | rs4646903* | 10 | Downstream, no info in F-SNP | Associated with breast cancer risk[^36^](#_ENREF_36)^,^ [^37^](#_ENREF_37) + endometrial cancer risk[^38^](#_ENREF_38) + meta-analysis prostate cancer risk[^39^](#_ENREF_39) |
|  | | *Not selected: MAF too low* | rs1048943 | 3 | NS coding, 0.13 | Associated with ovarian cancer risk meta-analysis[^40^](#_ENREF_40)^,^ [^41^](#_ENREF_41) + breast cancer risk[^42^](#_ENREF_42)^,^ [^43^](#_ENREF_43) + prostate cancer risk[^26^](#_ENREF_26) |
| ***CYP1A2*** | | Gene expression study[^15^](#_ENREF_15) |  |  |  |  |
|  | |  | rs762551* | 28 | Intronic, no info in F-SNP | Associated with breast cancer risk[^44-46^](#_ENREF_44) + ovarian cancer risk[^45^](#_ENREF_45)^,^ [^46^](#_ENREF_46) + risk of female cancers[^47^](#_ENREF_47) |
| ***CYP1B1*** | | Gene expression study[^5^](#_ENREF_5) |  |  |  |  |
|  | |  | rs1800440 | 19 | NS coding, 0.90 | Associated with endometrial cancer risk[^20^](#_ENREF_20)^,^ [^34^](#_ENREF_34)^,^ [^48^](#_ENREF_48) |
|  | |  | rs1056827* | 36 | NS coding, 0.75 | Associated with breast cancer risk[^31^](#_ENREF_31) + prostate cancer risk[^26^](#_ENREF_26)^,^ [^49^](#_ENREF_49) |
|  | |  | rs1056836* | 44 | NS coding, 0.86 | Associated with ovarian cancer risk[^50^](#_ENREF_50)^,^ [^51^](#_ENREF_51) + endometrial cancer risk[^34^](#_ENREF_34) + increased prostate cancer risk[^52^](#_ENREF_52) |
| ***CYP2C19*** | | KEGG Pathway Steroid Hormone Biosynthesis |  |  |  |  |
|  | |  | rs4917623 | 49 | Intronic, 0.21 | Associated with breast cancer risk[^44^](#_ENREF_44) |
| ***CYP3A4*** | | KEGG Pathway Steroid Hormone Biosynthesis |  |  |  |  |
|  | | *Not selected: MAF too low* | rs10273424 | 9 | Intergenic, no info in F-SNP | Associated with breast cancer risk[^53^](#_ENREF_53) + prostate cancer risk[^54-56^](#_ENREF_54) |
| ***CYP11A1*** | | Gene expression studies[^12^](#_ENREF_12)^,^ [^57^](#_ENREF_57) |  |  |  |  |
|  | |  | rs7173655* | 33 | Intronic, no info in F-SNP | Associated with endometrial cancer risk[^58^](#_ENREF_58) |
|  | |  | rs4555110 | 16 | Intergenic, no info in F-SNP | Associated with endometrial cancer risk[^58^](#_ENREF_58) |
|  | |  | rs3825944* | 17 | Intergenic, 0.77 | Associated with endometrial cancer risk[^58^](#_ENREF_58) |
|  | |  | rs2959008* | 32 | Intronic, no info in F-SNP | Associated with breast cancer risk[^59^](#_ENREF_59) |
| ***CYP17A1*** | | Gene expression study[^57^](#_ENREF_57) |  |  |  |  |
|  | |  | rs743572* | 38 | 5 prime UTR, 0.05 | Associated with breast cancer risk[^44^](#_ENREF_44) + endometrial cancer risk[^60^](#_ENREF_60) + prostate cancer risk[^61^](#_ENREF_61) |
|  | |  | rs4919682* | 28 | Intergenic, 0.5 | Marginally associated increased breast cancer risk[^62^](#_ENREF_62) |
|  | |  | rs4919687* | 33 | Intronic, 0 | Marginally associated increased breast cancer risk[^62^](#_ENREF_62) |
| ***CYP19A1*** | | KEGG Pathway Steroid Hormone Biosynthesis |  |  |  |  |
|  | |  | rs4775936 | 47 | Intronic, 0 | Associated with endometrial cancer risk[^63^](#_ENREF_63) |
|  | |  | rs727479 | 32 | Intronic, 0.18 | Associated with endometrial cancer risk[^64^](#_ENREF_64) + ovarian cancer risk[^65^](#_ENREF_65) |
|  | |  | rs749292* | 46 | Intronic, 0.21 | Associated with increased endometrial cancer risk[^64^](#_ENREF_64) + increased ovarian cancer risk[^65^](#_ENREF_65) |
| ***HSD3B1/***  ***HSD3B2 cluster*** | | Gene expression study[^13^](#_ENREF_13)^,^ [^66^](#_ENREF_66) |  |  |  |  |
|  | |  | rs6203 | 39 | S coding, 0.33 | Associated with prostate cancer risk[^26^](#_ENREF_26) |
|  | |  | rs1047303* | 35 | NS coding, 0.92 | Associated with prostate cancer risk[^26^](#_ENREF_26)^,^ [^67^](#_ENREF_67) |
|  | |  | rs6428830 | 33 | Intronic, 0.10 | Associated with prostate cancer risk[^68^](#_ENREF_68) |
|  | |  | rs4659175* | 32 | 5’ near gene, 0.21 | No leads in HugeNavigator and PubMed (in Dec 2013) |
|  | |  | rs1538989* | 48 | Downstream, 0.10 | Associated with prostate cancer risk[^69^](#_ENREF_69) |
| ***HSD17B1*** | | KEGG Pathway Steroid Hormone Biosynthesis |  |  |  |  |
|  | |  | rs676387 | 34 | Intronic, 0.24 | Associated with breast cancer risk[^70^](#_ENREF_70) |
|  | |  | rs605059 | 43 | NS coding, 0.20 | Associated with breast cancer Yao 2010 + prostate cancer risk[^26^](#_ENREF_26) |
| ***HSD17B3*** | | Gene expression study[^13^](#_ENREF_13) |  |  |  |  |
|  | |  | rs2257157* | 45 | Intronic, 0.21 | Associated with prostate cancer progression[^71^](#_ENREF_71) |
|  | |  | rs2253502* | 20 | Intronic, 0.05 | Associated with prostate cancer risk[^32^](#_ENREF_32) |
| ***SHBG*** | | KEGG Pathway Steroid Hormone Biosynthesis |  |  |  |  |
|  | |  | rs6259* | 14 | NS coding, 0.5 | Associated with prostate cancer risk[^26^](#_ENREF_26)^,^ [^72^](#_ENREF_72) |
|  | |  | rs1799941 | 23 | Intronic, no info in F-SNP | Associated with ovarian cancer risk[^73^](#_ENREF_73) |
| ***SRD5A1*** | | Gene expression studies[^13^](#_ENREF_13) [^57^](#_ENREF_57) |  |  |  |  |
|  | |  | rs10076470 | 38 | Splice site, intronic, 1.0 | No leads in HugeNavigator and PubMed (in Dec 2013) |
|  | |  | rs3736316 | 38 | S coding, 0.11 | Modifies relationship between postmenopausal hormone treatment and postmenopausal breast cancer risk[^74^](#_ENREF_74) |
|  | |  | rs8192120* | 36 | Intronic, 0.27 | Associated with endometrial cancer risk[^29^](#_ENREF_29) |
|  | |  | rs824811* | 23 | Intronic, 0.22 | Associated with endometrial cancer risk[^29^](#_ENREF_29) |
| ***SRD5A2*** | | KEGG Pathway Steroid Hormone Biosynthesis |  |  |  |  |
|  | |  | rs523349 | 19 | NS coding, 0.33 | Associated with prostate cancer risk[^75^](#_ENREF_75) + prostate cancer risk meta-analysis[^76^](#_ENREF_76)^,^ [^77^](#_ENREF_77) |
|  | |  | rs623419 | 43 | Intergenic, 0 | Associated with prostate cancer risk[^78^](#_ENREF_78) |
| ***SULT1A1*** | | Gene expression study[^5^](#_ENREF_5) |  |  |  |  |
|  | | *Not selected: not possible to design primer* | rs9282861 | 26 | NS coding, 0.75 | Associated with ovarian cancer risk[^45^](#_ENREF_45) + risk of female cancers[^47^](#_ENREF_47)+ modifies association between endogenous hormones and BMI and breast cancer risk[^79^](#_ENREF_79) |
|  | |  | rs6839* | 28 | 3 prime UTR, no info in F-SNP | Associated with endometrial cancer risk[^38^](#_ENREF_38) |
|  | |  | rs1042157* | 34 | 3 prime UTR, no info in F-SNP | Associated with endometrial cancer risk[^38^](#_ENREF_38) |
| ***SULT1E1*** | | Gene expression study[^13^](#_ENREF_13) |  |  |  |  |
|  | |  | rs3736599* | 14 | 5 prime UTR, 0.5 | Associated with endometrial cancer risk[^80^](#_ENREF_80) |
| **UGT1A** | | Gene expression studies[^5^](#_ENREF_5)^,^ [^13^](#_ENREF_13)^,^ [^81^](#_ENREF_81) |  |  |  |  |
|  | |  | rs2070959* | 31 | NS coding, 0.25 | Associated with endometrial cancer risk[^82^](#_ENREF_82) |
|  | |  | rs10176426 | 12 | Intronic, 0.10 | Interaction with heterocyclic amines for prostate cancer risk[^83^](#_ENREF_83) |
|  | |  | rs4663335 | 16 | Intergenic, no info in F-SNP | Interaction with heterocyclic amines for prostate cancer risk[^83^](#_ENREF_83) |
|  | |  | rs6742078 | 31 | Intronic, 0.5 | Interaction with heterocyclic amines for prostate cancer risk[^83^](#_ENREF_83) |
| ***UGT2B17*** | | Gene expression study[^13^](#_ENREF_13) |  |  |  |  |
|  | | *Not selected: takes 3 SNPs to genotype* | Gene deletion | 12 |  | Associated with prostate cancer risk[^84-86^](#_ENREF_84) + breast cancer risk[^87^](#_ENREF_87) |
| ***ESR1*** | | Sex hormone nuclear receptor |  |  |  |  |
|  | |  | rs2077647 | 41 | S coding, 0.5 | Associated with breast cancer risk[^88^](#_ENREF_88)^,^ [^89^](#_ENREF_89) + endometrial cancer risk [^90^](#_ENREF_90) |
|  | |  | rs2234693* | 41 | Intronic, 0.21 | Associated with endometrial cancer risk[^91-94^](#_ENREF_91) + prostate cancer risk[^26^](#_ENREF_26)^,^ [^95^](#_ENREF_95) |
|  | |  | rs3798577 | 48 | 3 prime UTR, 0 | Increased breast cancer risk[^96^](#_ENREF_96) |
|  | |  | rs9340799 | 31 | Intronic, 0.18 | Associated with breast cancer risk[^97^](#_ENREF_97) + endometrial cancer risk[^91^](#_ENREF_91)^,^ [^94^](#_ENREF_94) + prostate cancer risk meta-analysis[^95^](#_ENREF_95)^,^ [^98^](#_ENREF_98) |
|  | |  | rs3020314 | 26 | Intronic, 0 | Associated with breast cancer risk[^99^](#_ENREF_99) + endometrial cancer risk meta-analysis[^92^](#_ENREF_92) |
| ***ESR2*** | | Sex hormone nuclear receptor |  |  |  |  |
|  | |  | rs4986938* | 38 | Intronic, 0 | Associated with breast risk[^100^](#_ENREF_100) + prostate cancer risk[^101^](#_ENREF_101) |
|  | |  | rs1256030 | 42 | Intronic, 0.18 | Associated with ovarian cancer[^102^](#_ENREF_102) |
|  | |  | rs2987983* | 31 | Intronic, 0.5 | Associated with breast cancer risk[^103^](#_ENREF_103) + prostate cancer risk[^104^](#_ENREF_104) |
| ***PGR*** | | Sex hormone nuclear receptor |  |  |  |  |
|  | | *Not selected: not possible to design primer* | rs3740753 | 26 | NS coding, 0.5 | Associated with ovarian cancer risk[^105^](#_ENREF_105) |
|  | |  | rs660149* | 25 | Intronic, 0.18 | Associated with breast cancer risk[^106^](#_ENREF_106) |
|  | |  | rs471767 | 31 | Downstream, 0.5 | Associated with endometrial cancer risk[^107^](#_ENREF_107) |
|  | |  | rs1042838 | 20 | NS coding, 0.56 | Associated with ovarian cancer risk[^108^](#_ENREF_108)^,^[^109^](#_ENREF_109)^,^ [^110^](#_ENREF_110) + breast cancer risk[^106^](#_ENREF_106)^,^ [^110^](#_ENREF_110)^,^ [^111^](#_ENREF_111) + endometrial cancer risk[^112^](#_ENREF_112) |
| ***AR*** | | Sex hormone nuclear receptor |  |  |  |  |
|  | |  | rs6152 | 18 | S coding, 0.33 | Associated with prostate cancer risk[^26^](#_ENREF_26)^,^ [^78^](#_ENREF_78) + metastatic prostate cancer risk[^113^](#_ENREF_113) + endometrial cancer risk[^114^](#_ENREF_114) |
|  | |  | rs7061037 | 19 | Intronic, 0.21 | Associated with prostate cancer risk[^78^](#_ENREF_78) |
|  | |  | rs5964607 | 21 | Intergenic, no info in F-SNP | Associated with prostate cancer risk[^78^](#_ENREF_78) |
|  | |  | rs5919393 | 17 | Intronic, 0.05 | Associated with endometrial cancer risk[^29^](#_ENREF_29) |
| ***Category 2b: Oxidative stress*** | | | | | | |
| ***CAT*** | | Gene expression study[^57^](#_ENREF_57) |  |  |  |  |
|  | |  | rs4756146 | 8 | Intronic, 0.18 | Associated with postmenopausal breast cancer risk[^115^](#_ENREF_115) |
|  | |  | rs1001179* | 25 | 5’ near gene, 0.24 | Associated with prostate cancer risk[^116^](#_ENREF_116)^,^ [^117^](#_ENREF_117) + interaction with fruits + vegetables for breast cancer risk[^118^](#_ENREF_118) + interaction with postmenopausal hormone treatment[^119^](#_ENREF_119) |
|  | |  | rs511895* | 41 | Intronic, 0.21 | Associated with breast cancer risk[^120^](#_ENREF_120) |
|  | |  | rs554518 | 15 | Upstream, no info in F-SNP | Associated with prostate cancer risk (borderline sign.)[^121^](#_ENREF_121) |
| ***GPX1*** | | Gene expression study[^21^](#_ENREF_21) |  |  |  |  |
|  | |  | rs1050450 | 19 | NS coding, 0.54 | Associated with breast cancer risk[^122^](#_ENREF_122)^,^ [^123^](#_ENREF_123) + prostate cancer risk[^116^](#_ENREF_116)^,^ [^124^](#_ENREF_124) + interaction with selenium for prostate cancer risk[^125^](#_ENREF_125) |
|  | |  | rs3448* | 27 | 3-UTR, 0.30 | Associated with prostate cancer risk[^126^](#_ENREF_126) |
| ***CuZnSOD (SOD1)*** | | Gene expression studies[^57^](#_ENREF_57)^,^ [^81^](#_ENREF_81)^,^[^127^](#_ENREF_127) |  |  |  |  |
|  | |  | rs10432782* | 12 | Intronic, 0.21 | Interaction with selenium for prostate cancer risk[^128^](#_ENREF_128) |
| ***MnSOD (SOD2)*** | | Gene expression study[^127^](#_ENREF_127) |  |  |  |  |
|  | |  | rs4880* | 45 | NS coding, 0.33 | Associated with breast cancer risk[^123^](#_ENREF_123)^,^ [^129-131^](#_ENREF_129) + prostate cancer risk[^131-133^](#_ENREF_131) + ovarian cancer risk [^134^](#_ENREF_134) |
|  | |  | rs5746136* | 33 | UTR-3, 0.18 | Associated with prostate cancer risk[^121^](#_ENREF_121) |
|  | |  | rs2758330 | 25 | Intronic, 0 | Interaction with selenium for prostate cancer risk[^128^](#_ENREF_128) |
| ***TXN*** | | Gene expression studies[^5^](#_ENREF_5)^,^ [^12^](#_ENREF_12) |  |  |  |  |
|  | |  | rs2301241* | 41 | Upstream, no info in F-SNP | Associated with breast cancer risk[^135^](#_ENREF_135) |
| ***NQO1*** | | Gene expression studies[^5^](#_ENREF_5)^,^ [^13^](#_ENREF_13) |  |  |  |  |
|  | |  | rs1800566* | 22 | NS coding, 1 | Associated with breast cancer risk meta-analysis[^136^](#_ENREF_136) + prostate cancer risk[^26^](#_ENREF_26)^,^ [^137^](#_ENREF_137) + modifies association between oral contraceptives and breast cancer risk[^138^](#_ENREF_138) |
| ***Category 2c: DNA damage repair*** | | | | | | |
| ***OGG1*** | | Acrylamide-Hb adducts associated with urinary 8-OHdG adducts in a cross-sectional study in humans.[^139^](#_ENREF_139) OGG1 is a base excision repair gene that removes these adducts. |  |  |  |  |
|  | |  | rs1052133* | 22 | NS coding, 0.29 | Associated with breast cancer risk meta-analysis[^140^](#_ENREF_140) + endometrial cancer risk[^141^](#_ENREF_141) + prostate cancer risk meta-analysis[^142^](#_ENREF_142) |
| ***XRCC1*** | | Polymorphism study[^143^](#_ENREF_143) |  |  |  |  |
|  | | Polymorphism study[^143^](#_ENREF_143) | rs25487* | 37 | NS coding, 0.41 | Associated with breast cancer risk[^144^](#_ENREF_144)^,^ [^145^](#_ENREF_145) + prostate cancer risk[^146^](#_ENREF_146) + endometrial cancer risk[^141^](#_ENREF_141)^,^ [^147^](#_ENREF_147) |
| ***PCNA*** | | Gene expression study[^57^](#_ENREF_57) |  |  |  |  |
|  | |  | rs3626 | 12 | 3-UTR, no info in F-SNP | No leads in HugeNavigator and PubMed (in Dec 2013) |
|  | |  | rs25406 | 40 | Intronic, no info in F-SNP | No leads in HugeNavigator and PubMed (in Dec 2013) |
| ***MUTYH*** | | Polymorphism study[^143^](#_ENREF_143) |  |  |  |  |
|  | | Polymorphism study[^143^](#_ENREF_143) | rs3219489* | 28 | NS coding, 0.59 | No leads in HugeNavigator and PubMed (in Dec 2013) |
| ***XPC*** | | Polymorphism study[^143^](#_ENREF_143) |  |  |  |  |
|  | | Polymorphism study[^143^](#_ENREF_143) | rs2228000* | 29 | NS coding, 0.5 | Associated with breast cancer risk meta-analysis[^148^](#_ENREF_148) + endometrial cancer[^149^](#_ENREF_149) |
|  | | Polymorphism study[^143^](#_ENREF_143) | rs2228001* | 41 | NS coding, 0.88 | Associated with endometrial cancer[^149^](#_ENREF_149) |
| ***Category 2d: Other*** | | | | | | |
| ***CYP7B1*** | | Gene expression study[^13^](#_ENREF_13) |  |  |  |  |
|  | |  | rs7842714 | 47 | Intronic, 0.10 | No leads in HugeNavigator and PubMed (in Dec 2013) |
|  | |  | rs656506 | 46 | Upstream, 0.5 | No leads in HugeNavigator and PubMed (in Dec 2013) |
| ***STAR*** | | Gene expression study[^57^](#_ENREF_57) |  |  |  |  |
|  | |  | rs6474491 | 19 | Intergenic, no info in F-SNP | No leads in HugeNavigator and PubMed (in Dec 2013) |
|  | |  | rs3990403 | 22 | Downstream, no info in F-SNP | No leads in HugeNavigator and PubMed (in Dec 2013) |
| ***TSPO*** | | Gene expression study[^57^](#_ENREF_57) |  |  |  | Associated with poor breast cancer survival[^150^](#_ENREF_150) + prostate cancer progression[^151^](#_ENREF_151) |
|  | |  | rs6971 | 29 | NS coding, 0.83 | No leads in HugeNavigator and PubMed (in Dec 2013) |
| ***RRM2*** | | Gene expression study[^5^](#_ENREF_5) |  |  |  |  |
|  | |  | rs6759180* | 29 | Intronic, no info in F-SNP | Associated with breast cancer risk[^152^](#_ENREF_152) |
|  | |  | rs6741290* | 42 | Intronic, no info in F-SNP | Associated with breast cancer risk[^152^](#_ENREF_152) |
| ***SLC7A11*** | | Gene expression studies[^153^](#_ENREF_153)^,^[^5^](#_ENREF_5)^,^ [^154^](#_ENREF_154) |  |  |  |  |
|  | |  | rs6838248* | 43 | S coding, 0.37 | No leads in HugeNavigator and PubMed (in Dec 2013) |
| ***NFKB1*** | | Gene expression studies[^5^](#_ENREF_5)^,^ [^155^](#_ENREF_155) |  |  |  |  |
|  | |  | rs28362491*  (-94 ins/del ATTG) | 30 | 5’ near gene, 0.10 | Associated with ovarian cancer risk[^156^](#_ENREF_156)^,^ [^157^](#_ENREF_157) + prostate cancer risk[^158^](#_ENREF_158)^,^ [^159^](#_ENREF_159) |
| ***PTGS2*** | | Gene expression and activity studies[^160^](#_ENREF_160)^,^ [^161^](#_ENREF_161) |  |  |  |  |
|  | |  | rs5275* | 38 | 3-UTR, 0.18 | Associated with breast cancer risk[^162-164^](#_ENREF_162) |
| ***NOS2*** | | Gene expression and activity studies[^160^](#_ENREF_160)^,^ [^165^](#_ENREF_165) |  |  |  |  |
|  | |  | rs9282801 | 33 | Intronic, 0.21 | Associated with prostate cancer risk[^166^](#_ENREF_166) |
|  | |  | rs944722* | 49 | Intronic, not in F-SNP | Associated with prostate cancer risk[^166^](#_ENREF_166) |
| ***MGC12965*** | |  |  |  |  |  |
|  | | Kleinjans GWAS (personal communication) | rs1280350* | 19 | Intergenic, 0.51 | No leads in HugeNavigator and PubMed (in Dec 2013) |

* eventually genotyped

1. Duale N, Bjellaas T, Alexander J, et al. Biomarkers of human exposure to acrylamide and relation to polymorphisms in metabolizing genes. Toxicol Sci 2009; 108(1): 90-9.

2. Yang J, Qian LX, Wu HF, et al. Genetic polymorphisms in the cytochrome P450 1A1 and 2E1 genes, smoking, drinking and prostate cancer susceptibility: a case-control study in a Han nationality population in Southern China. Int J Urol 2006; 13(6): 773-80.

3. Wu SH, Tsai SM, Hou MF, et al. Interaction of genetic polymorphisms in cytochrome P450 2E1 and glutathione S-transferase M1 to breast cancer in Taiwanese woman without smoking and drinking habits. Breast Cancer Res Treat 2006; 100(1): 93-8.

4. Ferreira PM, Medeiros R, Vasconcelos A, et al. Association between CYP2E1 polymorphisms and susceptibility to prostate cancer. Eur J Cancer Prev 2003; 12(3): 205-11.

5. Clement FC, Dip R, Naegeli H. Expression profile of human cells in culture exposed to glycidamide, a reactive metabolite of the heat-induced food carcinogen acrylamide. Toxicology 2007; 240(1-2): 111-24.

6. Huang YF, Chen ML, Liou SH, Chen MF, Uang SN, Wu KY. Association of CYP2E1, GST and mEH genetic polymorphisms with urinary acrylamide metabolites in workers exposed to acrylamide. Toxicol Lett 2011.

7. Spurdle AB, Purdie DM, Webb PM, Chen X, Green A, Chenevix-Trench G. The microsomal epoxide hydrolase Tyr113His polymorphism: association with risk of ovarian cancer. Mol Carcinog 2001; 30(1): 71-8.

8. Goode EL, White KL, Vierkant RA, et al. Xenobiotic-Metabolizing gene polymorphisms and ovarian cancer risk. Mol Carcinog 2011; 50(5): 397-402.

9. Khedhaier A, Hassen E, Bouaouina N, Gabbouj S, Ahmed SB, Chouchane L. Implication of Xenobiotic Metabolizing Enzyme gene (CYP2E1, CYP2C19, CYP2D6, mEH and NAT2) polymorphisms in breast carcinoma. BMC Cancer 2008; 8: 109.

10. Mittal RD, Srivastava DL. Cytochrome P4501A1 and microsomal epoxide hydrolase gene polymorphisms: gene-environment interaction and risk of prostate cancer. DNA and cell biology 2007; 26(11): 791-8.

11. Pingarilho M, Oliveira NG, Martins C, et al. Induction of sister chromatid exchange by acrylamide and glycidamide in human lymphocytes: role of polymorphisms in detoxification and DNA-repair genes in the genotoxicity of glycidamide. Mutat Res 2013; 752(1-2): 1-7.

12. Yang HJ, Lee SH, Jin Y, et al. Toxicological effects of acrylamide on rat testicular gene expression profile. Reprod Toxicol 2005; 19(4): 527-34.

13. Mei N, Guo L, Tseng J, Dial SL, Liao W, Manjanatha MG. Gene expression changes associated with xenobiotic metabolism pathways in mice exposed to acrylamide. Environ Mol Mutagen 2008; 49(9): 741-5.

14. Kjuus H, Hansteen IL, Ryberg D, Goffeng LO, Ovrebo S, Skaug V. Chromosome aberrations in tunnel workers exposed to acrylamide and N-methylolacrylamide. Scand J Work Environ Health 2005; 31(4): 300-6.

15. Sen A, Ozgun O, Arinc E, Arslan S. Diverse action of acrylamide on cytochrome P450 and glutathione S-transferase isozyme activities, mRNA levels and protein levels in human hepatocarcinoma cells. Cell biology and toxicology 2012; 28(3): 175-86.

16. Qiu LX, Yuan H, Yu KD, et al. Glutathione S-transferase M1 polymorphism and breast cancer susceptibility: a meta-analysis involving 46,281 subjects. Breast Cancer Res Treat 2010; 121(3): 703-8.

17. Mo Z, Gao Y, Cao Y, Gao F, Jian L. An updating meta-analysis of the GSTM1, GSTT1, and GSTP1 polymorphisms and prostate cancer: a HuGE review. Prostate 2009; 69(6): 662-88.

18. Baxter SW, Thomas EJ, Campbell IG. GSTM1 null polymorphism and susceptibility to endometriosis and ovarian cancer. Carcinogenesis 2001; 22(1): 63-5.

19. Oliveira C, Lourenco GJ, Sagarra RA, Derchain SF, Segalla JG, Lima CS. Polymorphisms of glutathione S-transferase Mu 1 (GSTM1), Theta 1 (GSTT1), and Pi 1 (GSTP1) genes and epithelial ovarian cancer risk. Disease markers 2012; 33(3): 155-9.

20. Ashton KA, Proietto A, Otton G, et al. Polymorphisms in genes of the steroid hormone biosynthesis and metabolism pathways and endometrial cancer risk. Cancer epidemiology 2010; 34(3): 328-37.

21. Song J, Zhao M, Liu X, Zhu Y, Hu X, Chen F. Protection of cyanidin-3-glucoside against oxidative stress induced by acrylamide in human MDA-MB-231 cells. Food Chem Toxicol 2013; 58: 306-10.

22. Sergentanis TN, Economopoulos KP. GSTT1 and GSTP1 polymorphisms and breast cancer risk: a meta-analysis. Breast Cancer Res Treat 2010; 121(1): 195-202.

23. Cai Q, Wu T, Zhang W, et al. Genetic polymorphisms in glutathione S-transferases P1 (GSTP1) Ile105Val and prostate cancer risk: a systematic review and meta-analysis. Tumour Biol 2013; 34(6): 3913-22.

24. Chan QK, Khoo US, Ngan HY, et al. Single nucleotide polymorphism of pi-class glutathione s-transferase and susceptibility to endometrial carcinoma. Clin Cancer Res 2005; 11(8): 2981-5.

25. Mittal RD, Srivastava DS, Mandhani A, Kumar A, Mittal B. Polymorphism of GSTM1 and GSTT1 genes in prostate cancer: a study from North India. Indian journal of cancer 2004; 41(3): 115-9.

26. Cunningham JM, Hebbring SJ, McDonnell SK, et al. Evaluation of genetic variations in the androgen and estrogen metabolic pathways as risk factors for sporadic and familial prostate cancer. Cancer Epidemiol Biomarkers Prev 2007; 16(5): 969-78.

27. Chen XX, Zhao RP, Qiu LX, et al. Glutathione S-transferase T1 polymorphism is associated with breast cancer susceptibility. Cytokine 2011; 56(2): 477-80.

28. Karageorgi S, Prescott J, Wong JY, Lee IM, Buring JE, De Vivo I. GSTM1 and GSTT1 copy number variation in population-based studies of endometrial cancer risk. Cancer Epidemiol Biomarkers Prev 2011; 20(7): 1447-52.

29. Yang HP, Gonzalez Bosquet J, Li Q, et al. Common genetic variation in the sex hormone metabolic pathway and endometrial cancer risk: pathway-based evaluation of candidate genes. Carcinogenesis 2010; 31(5): 827-33.

30. Yu CC, Huang SP, Lee YC, et al. Molecular markers in sex hormone pathway genes associated with the efficacy of androgen-deprivation therapy for prostate cancer. PLoS One 2013; 8(1): e54627.

31. Reding KW, Li CI, Weiss NS, et al. Genetic variation in the progesterone receptor and metabolism pathways and hormone therapy in relation to breast cancer risk. Am J Epidemiol 2009; 170(10): 1241-9.

32. Kwon EM, Holt SK, Fu R, et al. Androgen metabolism and JAK/STAT pathway genes and prostate cancer risk. Cancer epidemiology 2012; 36(4): 347-53.

33. He XF, Wei W, Li SX, et al. Association between the COMT Val158Met polymorphism and breast cancer risk: a meta-analysis of 30,199 cases and 38,922 controls. Mol Biol Rep 2012; 39(6): 6811-23.

34. Teng Y, He C, Zuo X, Li X. Catechol-O-methyltransferase and cytochrome P-450 1B1 polymorphisms and endometrial cancer risk: a meta-analysis. Int J Gynecol Cancer 2013; 23(3): 422-30.

35. Ji Y, Olson J, Zhang J, et al. Breast cancer risk reduction and membrane-bound catechol O-methyltransferase genetic polymorphisms. Cancer Res 2008; 68(14): 5997-6005.

36. Moreno-Galvan M, Herrera-Gonzalez NE, Robles-Perez V, Velasco-Rodriguez JC, Tapia-Conyer R, Sarti E. Impact of CYP1A1 and COMT genotypes on breast cancer risk in Mexican women: a pilot study. The International journal of biological markers 2010; 25(3): 157-63.

37. Shin A, Kang D, Choi JY, et al. Cytochrome P450 1A1 (CYP1A1) polymorphisms and breast cancer risk in Korean women. Experimental & molecular medicine 2007; 39(3): 361-6.

38. Hirata H, Hinoda Y, Okayama N, et al. CYP1A1, SULT1A1, and SULT1E1 polymorphisms are risk factors for endometrial cancer susceptibility. Cancer 2008; 112(9): 1964-73.

39. Shaik AP, Jamil K, Das P. CYP1A1 polymorphisms and risk of prostate cancer: a meta-analysis. Urology journal 2009; 6(2): 78-86.

40. Sergentanis TN, Economopoulos KP, Choussein S, Vlahos NF. Cytochrome P450 1A1 (CYP1A1) gene polymorphisms and ovarian cancer risk: a meta-analysis. Mol Biol Rep 2012; 39(11): 9921-30.

41. Huang M, Chen Q, Xiao J, Zhao X, Liu C. CYP1A1 Ile462Val is a risk factor for ovarian cancer development. Cytokine 2012; 58(1): 73-8.

42. Sergentanis TN, Economopoulos KP. Four polymorphisms in cytochrome P450 1A1 (CYP1A1) gene and breast cancer risk: a meta-analysis. Breast Cancer Res Treat 2010; 122(2): 459-69.

43. Chen C, Huang Y, Li Y, Mao Y, Xie Y. Cytochrome P450 1A1 (CYP1A1) T3801C and A2455G polymorphisms in breast cancer risk: a meta-analysis. Journal of human genetics 2007; 52(5): 423-35.

44. Sangrajrang S, Sato Y, Sakamoto H, et al. Genetic polymorphisms of estrogen metabolizing enzyme and breast cancer risk in Thai women. Int J Cancer 2009; 125(4): 837-43.

45. Gulyaeva LF, Mikhailova ON, PustyInyak VO, et al. Comparative analysis of SNP in estrogen-metabolizing enzymes for ovarian, endometrial, and breast cancers in Novosibirsk, Russia. Adv Exp Med Biol 2008; 617: 359-66.

46. Tian Z, Li YL, Zhao L, Zhang CL. Role of CYP1A2 1F polymorphism in cancer risk: evidence from a meta-analysis of 46 case-control studies. Gene 2013; 524(2): 168-74.

47. Mikhailova ON, Gulyaeva LF, Prudnikov AV, Gerasimov AV, Krasilnikov SE. Estrogen-metabolizing gene polymorphisms in the assessment of female hormone-dependent cancer risk. The pharmacogenomics journal 2006; 6(3): 189-93.

48. McGrath M, Hankinson SE, Arbeitman L, Colditz GA, Hunter DJ, De Vivo I. Cytochrome P450 1B1 and catechol-O-methyltransferase polymorphisms and endometrial cancer susceptibility. Carcinogenesis 2004; 25(4): 559-65.

49. Cicek MS, Liu X, Casey G, Witte JS. Role of androgen metabolism genes CYP1B1, PSA/KLK3, and CYP11alpha in prostate cancer risk and aggressiveness. Cancer Epidemiol Biomarkers Prev 2005; 14(9): 2173-7.

50. Holt SK, Rossing MA, Malone KE, Schwartz SM, Weiss NS, Chen C. Ovarian cancer risk and polymorphisms involved in estrogen catabolism. Cancer Epidemiol Biomarkers Prev 2007; 16(3): 481-9.

51. Goodman MT, McDuffie K, Kolonel LN, et al. Case-control study of ovarian cancer and polymorphisms in genes involved in catecholestrogen formation and metabolism. Cancer Epidemiol Biomarkers Prev 2001; 10(3): 209-16.

52. Holt SK, Kwon EM, Fu R, et al. Association of variants in estrogen-related pathway genes with prostate cancer risk. Prostate 2013; 73(1): 1-10.

53. Johnson N, Walker K, Gibson LJ, et al. CYP3A variation, premenopausal estrone levels, and breast cancer risk. J Natl Cancer Inst 2012; 104(9): 657-69.

54. Rodrigues IS, Kuasne H, Losi-Guembarovski R, et al. Evaluation of the influence of polymorphic variants CYP1A1 2B, CYP1B1 2, CYP3A4 1B, GSTM1 0, and GSTT1 0 in prostate cancer. Urologic oncology 2011; 29(6): 654-63.

55. Zeigler-Johnson C, Friebel T, Walker AH, et al. CYP3A4, CYP3A5, and CYP3A43 genotypes and haplotypes in the etiology and severity of prostate cancer. Cancer Res 2004; 64(22): 8461-7.

56. Plummer SJ, Conti DV, Paris PL, Curran AP, Casey G, Witte JS. CYP3A4 and CYP3A5 genotypes, haplotypes, and risk of prostate cancer. Cancer Epidemiol Biomarkers Prev 2003; 12(9): 928-32.

57. Camacho L, Latendresse JR, Muskhelishvili L, et al. Effects of acrylamide exposure on serum hormones, gene expression, cell proliferation, and histopathology in male reproductive tissues of Fischer 344 rats. Toxicology letters 2012; 211(2): 135-43.

58. Terry K, McGrath M, Lee IM, Buring J, De Vivo I. Genetic variation in CYP11A1 and StAR in relation to endometrial cancer risk. Gynecol Oncol 2010; 117(2): 255-9.

59. Sun M, Yang X, Ye C, et al. Risk-association of CYP11A1 polymorphisms and breast cancer among Han Chinese women in Southern China. International journal of molecular sciences 2012; 13(4): 4896-905.

60. Olson SH, Orlow I, Bayuga S, et al. Variants in hormone biosynthesis genes and risk of endometrial cancer. Cancer Causes Control 2008; 19(9): 955-63.

61. Antognelli C, Mearini L, Talesa VN, Giannantoni A, Mearini E. Association of CYP17, GSTP1, and PON1 polymorphisms with the risk of prostate cancer. Prostate 2005; 63(3): 240-51.

62. Setiawan VW, Schumacher FR, Haiman CA, et al. CYP17 genetic variation and risk of breast and prostate cancer from the National Cancer Institute Breast and Prostate Cancer Cohort Consortium (BPC3). Cancer Epidemiol Biomarkers Prev 2007; 16(11): 2237-46.

63. Lundin E, Wirgin I, Lukanova A, et al. Selected polymorphisms in sex hormone-related genes, circulating sex hormones and risk of endometrial cancer. Cancer epidemiology 2012; 36(5): 445-52.

64. Setiawan VW, Doherty JA, Shu XO, et al. Two estrogen-related variants in CYP19A1 and endometrial cancer risk: a pooled analysis in the Epidemiology of Endometrial Cancer Consortium. Cancer Epidemiol Biomarkers Prev 2009; 18(1): 242-7.

65. Goodman MT, Lurie G, Thompson PJ, McDuffie KE, Carney ME. Association of two common single-nucleotide polymorphisms in the CYP19A1 locus and ovarian cancer risk. Endocr Relat Cancer 2008; 15(4): 1055-60.

66. Lee T, Manjanatha MG, Aidoo A, et al. Expression analysis of hepatic mitochondria-related genes in mice exposed to acrylamide and glycidamide. Journal of toxicology and environmental health Part A 2012; 75(6): 324-39.

67. Park JY, Tanner JP, Sellers TA, et al. Association between polymorphisms in HSD3B1 and UGT2B17 and prostate cancer risk. Urology 2007; 70(2): 374-9.

68. Setlur SR, Chen CX, Hossain RR, et al. Genetic variation of genes involved in dihydrotestosterone metabolism and the risk of prostate cancer. Cancer Epidemiol Biomarkers Prev 2010; 19(1): 229-39.

69. Beuten J, Gelfond JA, Franke JL, et al. Single and multigenic analysis of the association between variants in 12 steroid hormone metabolism genes and risk of prostate cancer. Cancer Epidemiol Biomarkers Prev 2009; 18(6): 1869-80.

70. Feigelson HS, Cox DG, Cann HM, et al. Haplotype analysis of the HSD17B1 gene and risk of breast cancer: a comprehensive approach to multicenter analyses of prospective cohort studies. Cancer Res 2006; 66(4): 2468-75.

71. Audet-Walsh E, Bellemare J, Lacombe L, et al. The impact of germline genetic variations in hydroxysteroid (17-beta) dehydrogenases on prostate cancer outcomes after prostatectomy. Eur Urol 2012; 62(1): 88-96.

72. Berndt SI, Chatterjee N, Huang WY, et al. Variant in sex hormone-binding globulin gene and the risk of prostate cancer. Cancer Epidemiol Biomarkers Prev 2007; 16(1): 165-8.

73. Garcia-Closas M, Brinton LA, Lissowska J, et al. Ovarian cancer risk and common variation in the sex hormone-binding globulin gene: a population-based case-control study. BMC Cancer 2007; 7: 60.

74. Hein R, Abbas S, Seibold P, Salazar R, Flesch-Janys D, Chang-Claude J. Polymorphism Thr160Thr in SRD5A1, involved in the progesterone metabolism, modifies postmenopausal breast cancer risk associated with menopausal hormone therapy. Breast Cancer Res Treat 2012; 131(2): 653-61.

75. Balistreri CR, Caruso C, Carruba G, Miceli V, Candore G. Genotyping of sex hormone-related pathways in benign and malignant human prostate tissues: data of a preliminary study. OMICS 2011; 15(6): 369-74.

76. Li X, Huang Y, Fu X, et al. Meta-analysis of three polymorphisms in the steroid-5-alpha-reductase, alpha polypeptide 2 gene (SRD5A2) and risk of prostate cancer. Mutagenesis 2011; 26(3): 371-83.

77. Wang C, Tao W, Chen Q, Hu H, Wen XY, Han R. SRD5A2 V89L polymorphism and prostate cancer risk: a meta-analysis. Prostate 2010; 70(2): 170-8.

78. Lindstrom S, Zheng SL, Wiklund F, et al. Systematic replication study of reported genetic associations in prostate cancer: Strong support for genetic variation in the androgen pathway. Prostate 2006; 66(16): 1729-43.

79. Yang G, Gao YT, Cai QY, Shu XO, Cheng JR, Zheng W. Modifying effects of sulfotransferase 1A1 gene polymorphism on the association of breast cancer risk with body mass index or endogenous steroid hormones. Breast Cancer Res Treat 2005; 94(1): 63-70.

80. Rebbeck TR, Troxel AB, Wang Y, et al. Estrogen sulfation genes, hormone replacement therapy, and endometrial cancer risk. J Natl Cancer Inst 2006; 98(18): 1311-20.

81. Hasegawa K, Miwa S, Isomura K, Tsutsumiuchi K, Taniguchi H, Miwa J. Acrylamide-responsive genes in the nematode Caenorhabditis elegans. Toxicol Sci 2007.

82. Deming SL, Zheng W, Xu WH, et al. UGT1A1 genetic polymorphisms, endogenous estrogen exposure, soy food intake, and endometrial cancer risk. Cancer Epidemiol Biomarkers Prev 2008; 17(3): 563-70.

83. Koutros S, Berndt SI, Sinha R, et al. Xenobiotic metabolizing gene variants, dietary heterocyclic amine intake, and risk of prostate cancer. Cancer Res 2009; 69(5): 1877-84.

84. Park J, Chen L, Ratnashinge L, et al. Deletion polymorphism of UDP-glucuronosyltransferase 2B17 and risk of prostate cancer in African American and Caucasian men. Cancer Epidemiol Biomarkers Prev 2006; 15(8): 1473-8.

85. Karypidis AH, Olsson M, Andersson SO, Rane A, Ekstrom L. Deletion polymorphism of the UGT2B17 gene is associated with increased risk for prostate cancer and correlated to gene expression in the prostate. The pharmacogenomics journal 2008; 8(2): 147-51.

86. Cai L, Huang W, Chou KC. Prostate cancer with variants in CYP17 and UGT2B17 genes: a meta-analysis. Protein and peptide letters 2012; 19(1): 62-9.

87. Eskandari-Nasab E, Hashemi M, Rezaei H, et al. Evaluation of UDP-glucuronosyltransferase 2B17 (UGT2B17) and dihydrofolate reductase (DHFR) genes deletion and the expression level of NGX6 mRNA in breast cancer. Mol Biol Rep 2012; 39(12): 10531-9.

88. Fernandez LP, Milne RL, Barroso E, et al. Estrogen and progesterone receptor gene polymorphisms and sporadic breast cancer risk: a Spanish case-control study. Int J Cancer 2006; 119(2): 467-71.

89. Hsiao WC, Young KC, Lin SL, Lin PW. Estrogen receptor-alpha polymorphism in a Taiwanese clinical breast cancer population: a case-control study. Breast Cancer Res 2004; 6(3): R180-6.

90. Sasaki M, Tanaka Y, Kaneuchi M, Sakuragi N, Dahiya R. Polymorphisms of estrogen receptor alpha gene in endometrial cancer. Biochem Biophys Res Commun 2002; 297(3): 558-64.

91. Ashton KA, Proietto A, Otton G, et al. Estrogen receptor polymorphisms and the risk of endometrial cancer. BJOG : an international journal of obstetrics and gynaecology 2009; 116(8): 1053-61.

92. Zhou X, Gu Y, Wang DN, Ni S, Yan J. Eight functional polymorphisms in the estrogen receptor 1 gene and endometrial cancer risk: a meta-analysis. PLoS One 2013; 8(4): e60851.

93. Wang Y, Cui M, Zheng L. Genetic polymorphisms in the estrogen receptor-alpha gene and the risk of endometrial cancer: a meta-analysis. Acta obstetricia et gynecologica Scandinavica 2012; 91(8): 911-6.

94. Wedren S, Lovmar L, Humphreys K, et al. Estrogen receptor alpha gene polymorphism and endometrial cancer risk--a case-control study. BMC Cancer 2008; 8: 322.

95. Wang YM, Liu ZW, Guo JB, Wang XF, Zhao XX, Zheng X. ESR1 Gene Polymorphisms and Prostate Cancer Risk: A HuGE Review and Meta-Analysis. PLoS One 2013; 8(6): e66999.

96. Zhang L, Gu L, Qian B, et al. Association of genetic polymorphisms of ER-alpha and the estradiol-synthesizing enzyme genes CYP17 and CYP19 with breast cancer risk in Chinese women. Breast Cancer Res Treat 2009; 114(2): 327-38.

97. Wang J, Higuchi R, Modugno F, et al. Estrogen receptor alpha haplotypes and breast cancer risk in older Caucasian women. Breast Cancer Res Treat 2007; 106(2): 273-80.

98. Ding X, Cui FM, Xu ST, et al. Variants on ESR1 and their association with prostate cancer risk: a meta-analysis. Asian Pacific journal of cancer prevention : APJCP 2012; 13(8): 3931-6.

99. Mavaddat N, Dunning AM, Ponder BA, Easton DF, Pharoah PD. Common genetic variation in candidate genes and susceptibility to subtypes of breast cancer. Cancer Epidemiol Biomarkers Prev 2009; 18(1): 255-9.

100. Yu KD, Rao NY, Chen AX, Fan L, Yang C, Shao ZM. A systematic review of the relationship between polymorphic sites in the estrogen receptor-beta (ESR2) gene and breast cancer risk. Breast Cancer Res Treat 2011; 126(1): 37-45.

101. Safarinejad MR, Safarinejad S, Shafiei N, Safarinejad S. Estrogen receptors alpha (rs2234693 and rs9340799), and beta (rs4986938 and rs1256049) genes polymorphism in prostate cancer: evidence for association with risk and histopathological tumor characteristics in Iranian men. Mol Carcinog 2012; 51 Suppl 1: E104-17.

102. Lurie G, Wilkens LR, Thompson PJ, et al. Genetic polymorphisms in the estrogen receptor beta (ESR2) gene and the risk of epithelial ovarian carcinoma. Cancer Causes Control 2009; 20(1): 47-55.

103. Treeck O, Elemenler E, Kriener C, et al. Polymorphisms in the promoter region of ESR2 gene and breast cancer susceptibility. J Steroid Biochem Mol Biol 2009; 114(3-5): 207-11.

104. Thellenberg-Karlsson C, Lindstrom S, Malmer B, et al. Estrogen receptor beta polymorphism is associated with prostate cancer risk. Clin Cancer Res 2006; 12(6): 1936-41.

105. Modugno F. Ovarian cancer and polymorphisms in the androgen and progesterone receptor genes: a HuGE review. Am J Epidemiol 2004; 159(4): 319-35.

106. Pooley KA, Healey CS, Smith PL, et al. Association of the progesterone receptor gene with breast cancer risk: a single-nucleotide polymorphism tagging approach. Cancer Epidemiol Biomarkers Prev 2006; 15(4): 675-82.

107. Xu WH, Long JR, Zheng W, et al. Association of the progesterone receptor gene with endometrial cancer risk in a Chinese population. Cancer 2009; 115(12): 2693-700.

108. Pearce CL, Wu AH, Gayther SA, et al. Progesterone receptor variation and risk of ovarian cancer is limited to the invasive endometrioid subtype: results from the Ovarian Cancer Association Consortium pooled analysis. Br J Cancer 2008; 98(2): 282-8.

109. Leite DB, Junqueira MG, de Carvalho CV, et al. Progesterone receptor (PROGINS) polymorphism and the risk of ovarian cancer. Steroids 2008; 73(6): 676-80.

110. Rockwell LC, Rowe EJ, Arnson K, et al. Worldwide distribution of allelic variation at the progesterone receptor locus and the incidence of female reproductive cancers. American journal of human biology : the official journal of the Human Biology Council 2012; 24(1): 42-51.

111. Johnatty SE, Spurdle AB, Beesley J, et al. Progesterone receptor polymorphisms and risk of breast cancer: results from two Australian breast cancer studies. Breast Cancer Res Treat 2008; 109(1): 91-9.

112. Junqueira MG, da Silva ID, Nogueira-de-Souza NC, et al. Progesterone receptor (PROGINS) polymorphism and the risk of endometrial cancer development. Int J Gynecol Cancer 2007; 17(1): 229-32.

113. Hayes VM, Severi G, Eggleton SA, et al. The E211 G>A androgen receptor polymorphism is associated with a decreased risk of metastatic prostate cancer and androgenetic alopecia. Cancer Epidemiol Biomarkers Prev 2005; 14(4): 993-6.

114. Yang HP, Garcia-Closas M, Lacey JV, Jr., et al. Genetic variation in the androgen receptor gene and endometrial cancer risk. Cancer Epidemiol Biomarkers Prev 2009; 18(2): 585-9.

115. McCullough LE, Santella RM, Cleveland RJ, et al. Polymorphisms in oxidative stress genes, physical activity, and breast cancer risk. Cancer Causes Control 2012; 23(12): 1949-58.

116. Karunasinghe N, Han DY, Goudie M, et al. Prostate disease risk factors among a New Zealand cohort. Journal of nutrigenetics and nutrigenomics 2012; 5(6): 339-51.

117. Tefik T, Kucukgergin C, Sanli O, Oktar T, Seckin S, Ozsoy C. Manganese superoxide dismutase Ile58Thr, catalase C-262T and myeloperoxidase G-463A gene polymorphisms in patients with prostate cancer: relation to advanced and metastatic disease. BJU Int 2013; 112(4): E406-14.

118. Li Y, Ambrosone CB, McCullough MJ, et al. Oxidative stress-related genotypes, fruit and vegetable consumption and breast cancer risk. Carcinogenesis 2009; 30(5): 777-84.

119. Quick SK, Shields PG, Nie J, et al. Effect modification by catalase genotype suggests a role for oxidative stress in the association of hormone replacement therapy with postmenopausal breast cancer risk. Cancer Epidemiol Biomarkers Prev 2008; 17(5): 1082-7.

120. Cebrian A, Pharoah PD, Ahmed S, et al. Tagging single-nucleotide polymorphisms in antioxidant defense enzymes and susceptibility to breast cancer. Cancer Res 2006; 66(2): 1225-33.

121. Ding G, Liu F, Shen B, Feng C, Xu J, Ding Q. The association between polymorphisms in prooxidant or antioxidant enzymes (myeloperoxidase, SOD2, and CAT) and genes and prostate cancer risk in the Chinese population of Han nationality. Clinical genitourinary cancer 2012; 10(4): 251-5.

122. Ravn-Haren G, Olsen A, Tjonneland A, et al. Associations between GPX1 Pro198Leu polymorphism, erythrocyte GPX activity, alcohol consumption and breast cancer risk in a prospective cohort study. Carcinogenesis 2006; 27(4): 820-5.

123. Tsai SM, Wu SH, Hou MF, Chen YL, Ma H, Tsai LY. Oxidative stress-related enzyme gene polymorphisms and susceptibility to breast cancer in non-smoking, non-alcohol-consuming Taiwanese women: a case-control study. Annals of clinical biochemistry 2012; 49(Pt 2): 152-8.

124. Arsova-Sarafinovska Z, Matevska N, Eken A, et al. Glutathione peroxidase 1 (GPX1) genetic polymorphism, erythrocyte GPX activity, and prostate cancer risk. International urology and nephrology 2009; 41(1): 63-70.

125. Steinbrecher A, Meplan C, Hesketh J, et al. Effects of selenium status and polymorphisms in selenoprotein genes on prostate cancer risk in a prospective study of European men. Cancer Epidemiol Biomarkers Prev 2010; 19(11): 2958-68.

126. Geybels MS, van den Brandt PA, Schouten LJ, et al. Selenoprotein gene variants, toenail selenium levels, and risk for advanced prostate cancer. J Natl Cancer Inst 2014; 106(3): dju003.

127. Sadek IA. Short-term studies of the effect of acrylamide on the testes of the Egyptian toad. Folia morphologica 1989; 37(4): 427-30.

128. Abe M, Xie W, Regan MM, et al. Single-nucleotide polymorphisms within the antioxidant defence system and associations with aggressive prostate cancer. BJU Int 2011; 107(1): 126-34.

129. Tamimi RM, Hankinson SE, Spiegelman D, Colditz GA, Hunter DJ. Manganese superoxide dismutase polymorphism, plasma antioxidants, cigarette smoking, and risk of breast cancer. Cancer Epidemiol Biomarkers Prev 2004; 13(6): 989-96.

130. Cai Q, Shu XO, Wen W, et al. Genetic polymorphism in the manganese superoxide dismutase gene, antioxidant intake, and breast cancer risk: results from the Shanghai Breast Cancer Study. Breast Cancer Res 2004; 6(6): R647-55.

131. Bica CG, de Moura da Silva LL, Toscani NV, et al. MnSOD gene polymorphism association with steroid-dependent cancer. Pathol Oncol Res 2009; 15(1): 19-24.

132. Kang D, Lee KM, Park SK, et al. Functional variant of manganese superoxide dismutase (SOD2 V16A) polymorphism is associated with prostate cancer risk in the prostate, lung, colorectal, and ovarian cancer study. Cancer Epidemiol Biomarkers Prev 2007; 16(8): 1581-6.

133. Mao C, Qiu LX, Zhan P, et al. MnSOD Val16Ala polymorphism and prostate cancer susceptibility: a meta-analysis involving 8,962 subjects. Journal of cancer research and clinical oncology 2010; 136(7): 975-9.

134. Olson SH, Carlson MD, Ostrer H, et al. Genetic variants in SOD2, MPO, and NQO1, and risk of ovarian cancer. Gynecol Oncol 2004; 93(3): 615-20.

135. Seibold P, Hein R, Schmezer P, et al. Polymorphisms in oxidative stress-related genes and postmenopausal breast cancer risk. Int J Cancer 2011; 129(6): 1467-76.

136. Yuan W, Xu L, Chen W, et al. Evidence on the association between NQO1 Pro187Ser polymorphism and breast cancer risk in the current studies: a meta-analysis. Breast Cancer Res Treat 2011; 125(2): 467-72.

137. Mandal RK, Nissar K, Mittal RD. Genetic variants in metabolizing genes NQO1, NQO2, MTHFR and risk of prostate cancer: a study from North India. Mol Biol Rep 2012; 39(12): 11145-52.

138. Fowke JH, Shu XO, Dai Q, et al. Oral contraceptive use and breast cancer risk: modification by NAD(P)H:quinone oxoreductase (NQO1) genetic polymorphisms. Cancer Epidemiol Biomarkers Prev 2004; 13(8): 1308-15.

139. Lin CY, Lee HL, Chen YC, et al. Positive association between urinary levels of 8-hydroxydeoxyguanosine and the acrylamide metabolite N-acetyl-S-(propionamide)-cysteine in adolescents and young adults. J Hazard Mater 2013; 261: 372-7.

140. Yuan W, Xu L, Feng Y, et al. The hOGG1 Ser326Cys polymorphism and breast cancer risk: a meta-analysis. Breast Cancer Res Treat 2010; 122(3): 835-42.

141. Cincin ZB, Iyibozkurt AC, Kuran SB, Cakmakoglu B. DNA repair gene variants in endometrial carcinoma. Medical oncology 2012; 29(4): 2949-54.

142. Zhu S, Zhang H, Tang Y, Wang J. Polymorphisms in XPD and hOGG1 and prostate cancer risk: a meta-analysis. Urol Int 2012; 89(2): 233-40.

143. Pingarilho M, Oliveira NG, Martins C, et al. Genetic polymorphisms in detoxification and DNA repair genes and susceptibility to glycidamide-induced DNA damage. Journal of toxicology and environmental health Part A 2012; 75(13-15): 920-33.

144. Wu K, Su D, Lin K, Luo J, Au WW. XRCC1 Arg399Gln gene polymorphism and breast cancer risk: a meta-analysis based on case-control studies. Asian Pacific journal of cancer prevention : APJCP 2011; 12(9): 2237-43.

145. Huang Y, Li L, Yu L. XRCC1 Arg399Gln, Arg194Trp and Arg280His polymorphisms in breast cancer risk: a meta-analysis. Mutagenesis 2009; 24(4): 331-9.

146. Chen L, Ambrosone CB, Lee J, Sellers TA, Pow-Sang J, Park JY. Association between polymorphisms in the DNA repair genes XRCC1 and APE1, and the risk of prostate cancer in white and black Americans. The Journal of urology 2006; 175(1): 108-12; discussion 12.

147. Samulak D, Romanowicz-Makowska H, Smolarz B, Kulig A, Sporny S. Association between Arg399Gln polymorphism of X-ray repair cross-complementing 1 (XRCC1) gene and sporadic endometrial cancer in the Polish population. European journal of gynaecological oncology 2011; 32(5): 491-5.

148. He J, Shi TY, Zhu ML, Wang MY, Li QX, Wei QY. Associations of Lys939Gln and Ala499Val polymorphisms of the XPC gene with cancer susceptibility: a meta-analysis. Int J Cancer 2013; 133(8): 1765-75.

149. Weiss JM, Weiss NS, Ulrich CM, Doherty JA, Voigt LF, Chen C. Interindividual variation in nucleotide excision repair genes and risk of endometrial cancer. Cancer Epidemiol Biomarkers Prev 2005; 14(11 Pt 1): 2524-30.

150. Galiegue S, Casellas P, Kramar A, Tinel N, Simony-Lafontaine J. Immunohistochemical assessment of the peripheral benzodiazepine receptor in breast cancer and its relationship with survival. Clin Cancer Res 2004; 10(6): 2058-64.

151. Fafalios A, Akhavan A, Parwani AV, Bies RR, McHugh KJ, Pflug BR. Translocator protein blockade reduces prostate tumor growth. Clin Cancer Res 2009; 15(19): 6177-84.

152. Olson JE, Wang X, Goode EL, et al. Variation in genes required for normal mitosis and risk of breast cancer. Breast Cancer Res Treat 2010; 119(2): 423-30.

153. Ehlers A, Lenze D, Broll H, Zagon J, Hummel M, Lampen A. Dose dependent molecular effects of acrylamide and glycidamide in human cancer cell lines and human primary hepatocytes. Toxicol Lett 2013; 217(2): 111-20.

154. Hochstenbach K, van Leeuwen DM, Gmuender H, et al. Global gene expression analysis in cord blood reveals gender-specific differences in response to carcinogenic exposure in utero. Cancer epidemiology, biomarkers & prevention : a publication of the American Association for Cancer Research, cosponsored by the American Society of Preventive Oncology 2012; 21(10): 1756-67.

155. Shan X, Li Y, Meng X, Wang P, Jiang P, Feng Q. Curcumin and (-)-epigallocatechin-3-gallate attenuate acrylamide-induced proliferation in HepG2 cells. Food Chem Toxicol 2014; 66: 194-202.

156. Fan Y, Yu W, Ye P, et al. NFKB1 insertion/deletion promoter polymorphism increases the risk of advanced ovarian cancer in a Chinese population. DNA and cell biology 2011; 30(4): 241-5.

157. Huo ZH, Zhong HJ, Zhu YS, Xing B, Tang H. Roles of functional NFKB1 and beta-TrCP insertion/deletion polymorphisms in mRNA expression and epithelial ovarian cancer susceptibility. Genetics and molecular research : GMR 2013; 12(3): 3435-43.

158. Zhang P, Wei Q, Li X, et al. A functional insertion/deletion polymorphism in the promoter region of the NFKB1 gene increases susceptibility for prostate cancer. Cancer genetics and cytogenetics 2009; 191(2): 73-7.

159. Kopp TI, Friis S, Christensen J, Tjonneland A, Vogel U. Polymorphisms in genes related to inflammation, NSAID use, and the risk of prostate cancer among Danish men. Cancer genetics 2013; 206(7-8): 266-78.

160. Lyn-Cook LE, Jr., Tareke E, Word B, Starlard-Davenport A, Lyn-Cook BD, Hammons GJ. Food contaminant acrylamide increases expression of Cox-2 and nitric oxide synthase in breast epithelial cells. Toxicology and industrial health 2011; 27(1): 11-8.

161. Lim TG, Lee BK, Kwon JY, Jung SK, Lee KW. Acrylamide up-regulates cyclooxygenase-2 expression through the MEK/ERK signaling pathway in mouse epidermal cells. Food Chem Toxicol 2011; 49(6): 1249-54.

162. Langsenlehner U, Yazdani-Biuki B, Eder T, et al. The cyclooxygenase-2 (PTGS2) 8473T>C polymorphism is associated with breast cancer risk. Clin Cancer Res 2006; 12(4): 1392-4.

163. Fawzy MS, Aly NM, Shalaby SM, El-Sawy WH, Abdul-Maksoud RS. Cyclooxygenase-2 169C>G and 8473T>C gene polymorphisms and prostaglandin E2 level in breast cancer: a case-control study. Gene 2013; 527(2): 601-5.

164. Zhu W, Wei BB, Shan X, Liu P. -765G>C and 8473T>C polymorphisms of COX-2 and cancer risk: a meta-analysis based on 33 case-control studies. Mol Biol Rep 2010; 37(1): 277-88.

165. Kim K. Effect of subchronic acrylamide exposure on the expression of neuronal and inducible nitric oxide synthase in rat brain. J Biochem Mol Toxicol 2005; 19(3): 162-8.

166. Lee KM, Kang D, Park SK, et al. Nitric oxide synthase gene polymorphisms and prostate cancer risk. Carcinogenesis 2009; 30(4): 621-5.

| Supplemental Table 2: Description of SNPs and their genotype frequencies in the subcohort (participants with sample call rate ≥95% only) | | | | | | | | | | | | | |
| --- | --- | --- | --- | --- | --- | --- | --- | --- | --- | --- | --- | --- | --- |
|  | | | | | | | **Genotypes** | | | | | |  |
|  |  |  |  |  |  |  | **n** | | | **%** | | | **HWE†** |
| **SNP ID** | **Gene** | **Chr. Location** | **Ref. alleles wild type/variant** | **Minor Allele NLCS** | **MAF NLCS** | **SNPcall rate** | **11*** | **12*** | **22*** | **11** | **12** | **22** | **P value** |
| **Acrylamide metabolism** | | | | | | | | | | | | | |
| rs6413432 | *CYP2E1* | 10q26.3 | T/A | A | 9.0 | 99.9 | 1436 | 295 | 9 | 82.5 | 17.0 | 0.5 | 0.14 |
| rs915906 | *CYP2E1* | 10q26.3 | T/C | C | 14.9 | 99.9 | 1255 | 451 | 33 | 72.2 | 25.9 | 1.9 | 0.30 |
| rs2480258 | *CYP2E1* | 10q26.3 | G/A | A | 19.0 | 100 | 1129 | 563 | 49 | 64.8 | 32.3 | 2.8 | 0.03 |
| rs1051740 | *EPHX1* | 1q42.1 | T/C | C | 31.1 | 100 | 833 | 734 | 174 | 47.8 | 42.2 | 10.0 | 0.51 |
| rs4715354 | *GSTA5* | 6p12.2 | G/A | G | 48.4 | 100 | 458 | 879 | 404 | 26.3 | 50.5 | 23.2 | 0.65 |
| rs1695 | *GSTP1* | 11q13 | A/G | G | 35.9 | 100 | 706 | 821 | 214 | 40.6 | 47.2 | 12.3 | 0.30 |
| **Sex steroid metabolism** | | | | | | | | | | | | | |
| rs11252859 | *AKR1C1* | 10p15-p14 | C/T | T | 36.3 | 99.5 | 716 | 773 | 243 | 41.3 | 44.6 | 14.0 | 0.14 |
| rs7741 | *AKR1C2* | 10p15-p14 | G/A | G | 29.3 | 36.7 | 19 | 464 | 156 | 3.0 | 72.6 | 24.4 | <0.001 |
| rs11252887 | *AKR1C2* | 10p15-p14 | C/T | T | 28.3 | 99.3 | 874 | 695 | 135 | 51.3 | 40.8 | 7.9 | 0.85 |
| rs737865 | *COMT* | 22q11.21 | T/C | C | 25.8 | 100 | 953 | 678 | 110 | 54.7 | 38.9 | 6.3 | 0.47 |
| rs4646903 | *CYP1A1* | 15q24.1 | T/C | C | 8.3 | 98.3 | 1429 | 283 | 0 | 83.5 | 16.5 | 0.0 | <0.001 |
| rs2472299 | *CYP1A2* | 15q24.1 | G/A | A | 28.2 | 99.9 | 905 | 688 | 146 | 52.0 | 39.6 | 8.4 | 0.35 |
| rs1056827 | *CYP1B1* | 2p22.2 | G/T | T | 27.1 | 99.8 | 926 | 682 | 130 | 53.3 | 39.2 | 7.5 | 0.77 |
| rs1056836 | *CYP1B1* | 2p22.2 | C/G | G | 44.6 | 99.9 | 546 | 836 | 358 | 31.4 | 48.0 | 20.6 | 0.25 |
| rs3825944 | *CYP11A1* | 15q23-q24 | C/T | T | 16.5 | 100 | 1219 | 468 | 54 | 70.0 | 26.9 | 3.1 | 0.27 |
| rs2959008 | *CYP11A1* | 15q23-q24 | C/T | T | 33.8 | 99.9 | 764 | 774 | 201 | 43.9 | 44.5 | 11.6 | 0.82 |
| rs7173655 | *CYP11A1* | 15q23-q24 | T/C | C | 32.1 | 100 | 813 | 739 | 189 | 46.7 | 42.4 | 10.9 | 0.28 |
| rs4919682 | *CYP17A1* | 10q24.3 | C/T | T | 29.5 | 100 | 851 | 753 | 137 | 48.9 | 43.3 | 7.9 | 0.10 |
| rs4919687 | *CYP17A1* | 10q24.3 | G/A | A | 30.5 | 99.9 | 827 | 763 | 150 | 47.5 | 43.9 | 8.6 | 0.16 |
| rs743572 | *CYP17A1* | 10q24.3 | A/G | G | 39.9 | 99.9 | 625 | 843 | 272 | 35.9 | 48.4 | 15.6 | 0.66 |
| rs11632903 | *CYP19A1* | 15q21.1 | C/T | T | 44.3 | 100 | 546 | 846 | 349 | 31.4 | 48.6 | 20.0 | 0.52 |
| rs4659175 | *HSD3B1/B2* | 1p13.1 | C/T | T | 32.3 | 99.9 | 798 | 759 | 183 | 45.9 | 43.6 | 10.5 | 0.90 |
| rs1047303 | *HSD3B1/B2* | 1p13.1 | A/C | C | 31.6 | 99.9 | 800 | 782 | 158 | 46.0 | 44.9 | 9.1 | 0.09 |
| rs10923823 | *HSD3B1/B2* | 1p13.1 | C/T | C | 45.0 | 100 | 540 | 836 | 365 | 31.0 | 48.0 | 21.0 | 0.21 |
| rs6203 | *HSD3B1/B2* | 1p13.1 | C/T | T | 42.0 | 100 | 568 | 853 | 295 | 33.1 | 49.7 | 17.2 | 0.41 |
| rs6428830 | *HSD3B1/B2* | 1p13.1 | G/A | A | 29.8 | 99.9 | 833 | 743 | 139 | 48.6 | 43.3 | 8.1 | 0.13 |
| rs7546652 | *HSD3B1/B2* | 1p13.1 | T/C | C | 45.0 | 99.9 | 540 | 835 | 365 | 31.0 | 48.0 | 21.0 | 0.20 |
| rs2253502 | *HSD17B3* | 9q22 | T/C | C | 21.3 | 71.9 | 776 | 416 | 59 | 62.0 | 33.3 | 4.7 | 0.73 |
| rs2257157 | *HSD17B3* | 9q22 | T/C | C | 47.8 | 99.8 | 459 | 896 | 383 | 26.4 | 51.6 | 22.0 | 0.17 |
| rs6259 | *SHBG* | 17p13.1 | G/A | A | 11.2 | 99.4 | 1362 | 349 | 19 | 78.7 | 20.2 | 1.1 | 0.52 |
| rs8192120 | *SRD5A1* | 5p15 | C/A | A | 37.2 | 99.9 | 687 | 811 | 242 | 39.5 | 46.6 | 13.9 | 0.91 |
| rs824811 | *SRD5A1* | 5p15 | T/C | C | 23.2 | 100 | 1029 | 617 | 95 | 59.1 | 35.4 | 5.5 | 0.84 |
| rs1042157 | *SULT1A1* | 16p12.1 | C/T | T | 38.8 | 99.7 | 642 | 839 | 254 | 37.0 | 48.4 | 14.6 | 0.45 |
| rs6839 | *SULT1A1* | 16p12.1 | A/G | G | 34.6 | 99.3 | 765 | 730 | 234 | 44.2 | 42.2 | 13.5 | 0.005 |
| rs3736599 | *SULT1E1* | 4q13.1 | G/A | A | 9.5 | 99.9 | 1425 | 298 | 16 | 81.9 | 17.1 | 0.9 | 0.92 |
| rs2070959 | *UGT1A6-10* | 2q37 | A/G | G | 32.3 | 99.9 | 788 | 780 | 172 | 45.3 | 44.8 | 9.9 | 0.30 |
| **Nuclear receptors** | | | | | | | | | | | | | |
| rs2234693 | *ESR1* | 6q25.1 | T/C | C | 48.9 | 77.6 | 361 | 659 | 331 | 26.7 | 48.8 | 24.5 | 0.38 |
| rs2987983 | *ESR2* | 14q23.2 | T/C | C | 32.5 | 99.9 | 817 | 716 | 207 | 47.0 | 41.1 | 11.9 | 0.01 |
| rs4986938 | *ESR2* | 14q23.2 | G/A | A | 36.1 | 99.9 | 720 | 784 | 235 | 41.4 | 45.1 | 13.5 | 0.35 |
| rs660149 | *PGR* | 11q22-q23 | C/G | G | 26.0 | 100 | 950 | 675 | 116 | 54.6 | 38.8 | 6.7 | 0.79 |
| **Oxidative stress pathway** | | | | | | | | | | | | | |
| rs1001179 | *CAT* | 11p13 | G/A | A | 21.0 | 97.5 | 1074 | 535 | 89 | 63.3 | 31.5 | 5.2 | 0.04 |
| rs511895 | *CAT* | 11p13 | A/G | A | 41.3 | 100 | 584 | 875 | 282 | 33.5 | 50.3 | 16.2 | 0.13 |
| rs3448 | *GPX1* | 3p21.3 | C/T | T | 27.0 | 99.9 | 923 | 696 | 121 | 53.0 | 40.0 | 7.0 | 0.51 |
| rs1800566 | *NQO1* | 16q22.1 | C/T | T | 18.7 | 99.9 | 1146 | 538 | 56 | 65.9 | 30.9 | 3.2 | 0.46 |
| rs10432782 | *SOD1* | 21q22.11 | T/G | G | 11.4 | 100 | 1367 | 350 | 24 | 78.5 | 20.1 | 1.4 | 0.77 |
| rs4880 | *SOD2* | 6q25.3 | T/C | C | 49.1 | 100 | 445 | 882 | 414 | 25.6 | 50.7 | 23.8 | 0.57 |
| rs5746136 | *SOD2* | 6q25.3 | G/A | A | 29.6 | 100 | 872 | 708 | 161 | 50.1 | 40.7 | 9.2 | 0.32 |
| rs2301241 | *TXN* | 9q31 | C/T | C | 38.9 | 99.9 | 640 | 845 | 255 | 36.8 | 48.6 | 14.7 | 0.38 |
| **DNA repair** |  |  |  |  |  |  |  |  |  |  |  |  |  |
| rs1052133 | *OGG1* | 3p26.2 | C/G | G | 22.2 | 100 | 1055 | 600 | 86 | 60.6 | 34.5 | 4.9 | 0.95 |
| rs3219489 | *MUTYH* | 1p34.1 | G/C | C | 24.1 | 99.8 | 1005 | 628 | 104 | 57.9 | 36.2 | 6.0 | 0.65 |
| rs2228000 | *XPC* | 3p25 | C/T | T | 26.4 | 100 | 955 | 653 | 133 | 54.9 | 37.5 | 7.6 | 0.15 |
| rs2228001 | *XPC* | 3p25 | A/C | C | 39.3 | 100 | 633 | 846 | 262 | 36.4 | 48.6 | 15.0 | 0.45 |
| rs25487 | *XRCC1* | 19q13.2 | A/G | A | 35.6 | 99.9 | 721 | 799 | 220 | 41.4 | 45.9 | 12.6 | 0.95 |
| **Gene expression acrylamide/GWAS acrylamide/other** | | | | | | | | | | | | | |
| rs1280350 | *MGC12965* | 11q13.4 | G/T | T | 17.2 | 100 | 1195 | 494 | 52 | 68.6 | 28.4 | 3.0 | 0.91 |
| rs28362491 | *NFKB1* | 4q24 | ATTG/DEL | DEL | 40.2 | 99.9 | 633 | 815 | 291 | 36.4 | 46.9 | 16.7 | 0.30 |
| rs944722 | *NOS2* | 17q11.2-q12 | T/C | C | 39.1 | 96.7 | 646 | 757 | 280 | 38.4 | 45.0 | 16.6 | 0.02 |
| rs5275 | *PTGS2* | 1q25.2-q25.3 | T/C | C | 31.7 | 99.9 | 807 | 763 | 170 | 46.4 | 43.9 | 9.8 | 0.60 |
| rs6741290 | *RRM2* | 2p25-p24 | C/T | T | 42.7 | 99.9 | 576 | 842 | 321 | 33.1 | 48.4 | 18.5 | 0.67 |
| rs6759180 | *RRM2* | 2p25-p24 | A/G | G | 26.4 | 99.1 | 937 | 665 | 124 | 54.3 | 38.5 | 7.2 | 0.69 |
| rs6838248 | *SLC7A11* | 4q28-q32 | C/G | C | 46.6 | 99.9 | 394 | 835 | 511 | 22.6 | 48.0 | 29.4 | 0.13 |

* 11: wildtype/wildtype, 12: wildtype/variant, 22: variant/variant

† HWE = Hardy-Weinberg Equilibrium

Supplemental Table 3: other nominally (borderline) statistically significant interactions between SNPs and dietary acrylamide intake

on the risk of ovarian cancer and clear differences in acrylamide dose-responses between genotypes, 20.3 years of follow-up

|  | Acrylamide, continuous intake |  | Acrylamide, tertiles of intake | | | | | | | | Interaction | |  |
| --- | --- | --- | --- | --- | --- | --- | --- | --- | --- | --- | --- | --- | --- |
| SNP | 10 µg/day |  | N cases | Tertile 1 | N cases | Tertile 2 | N cases | Tertile 3 |  | P for trend | P for linear interaction | |  |
|  |  |  |  |  |  |  |  |  |  |  | Raw p | Benjamini-Hochberg adjusted p value |  |
| All |  |  |  |  |  |  |  |  |  |  |  |  |  |
| AKR1C1, rs11252859==0 | 1.17 (1.00-1.36) |  | 30 | Ref (1.00) | 29 | 0.93 (0.52-1.66) | 47 | 1.48 (0.86-2.53) |  | 0.14 | 0.09 | 0.66 |  |
| AKR1C1, rs11252859==1 | 1.02 (0.88-1.18) |  | 58 | Ref (1.00) | 35 | 0.70 (0.43-1.15) | 53 | 0.98 (0.63-1.55) |  | 0.97 |  |  |  |
| Never-smokers |  |  |  |  |  |  |  |  |  |  |  |  |  |
| AKR1C1, rs11252859==0 | 1.35 (1.09-1.68) |  | 18 | Ref (1.00) | 25 | 1.43 (0.70-2.91) | 29 | 1.96 (0.99-3.88) |  | 0.05 | 0.07 | 0.52 |  |
| AKR1C1, rs11252859==1 | 1.06 (0.88-1.26) |  | 34 | Ref (1.00) | 22 | 0.79 (0.42-1.47) | 35 | 1.11 (0.63-1.94) |  | 0.70 |  |  |  |
| All |  |  |  |  |  |  |  |  |  |  |  |  |  |
| CYP19A1, rs11632903=0 | 1.33 (1.04-1.70) |  | 22 | Ref (1.00) | 22 | 1.15 (0.59-2.25) | 34 | 1.66 (0.86-3.21) |  | 0.13 | 0.07 | 0.59 |  |
| CYP19A1, rs11632903==1 | 1.02 (0.89-1.16) |  | 69 | Ref (1.00) | 40 | 0.65 (0.42-1.01) | 66 | 0.95 (0.64-1.42) |  | 0.87 |  |  |  |
| Never-smokers |  |  |  |  |  |  |  |  |  |  |  |  |  |
| CYP19A1, rs11632903==0 | 1.42 (1.05-1.93) |  | 12 | Ref (1.00) | 15 | 1.20 (0.50-2.86) | 22 | 1.51 (0.65-3.54) |  | 0.34 | 0.09 | 0.53 |  |
| CYP19A1, rs11632903==1 | 1.07 (0.90-1.27) |  | 42 | Ref (1.00)) | 30 | 0.83 (0.49-1.41) | 42 | 1.09 (0.67-1.78) |  | 0.71 |  |  |  |
| All |  |  |  |  |  |  |  |  |  |  |  |  |  |
| GPX1, rs3448==0 | 1.16 (1.01-1.33) |  | 42 | Ref (1.00) | 31 | 0.87 (0.52-1.47) | 50 | 1.39 (0.87-2.22) |  | 0.18 | 0.30 | 0.76 |  |
| GPX1, rs3448==1 | 0.99 (0.82-1.20) |  | 46 | Ref (1.00) | 33 | 0.71 (0.42-1.18) | 50 | 0.87 (0.53-1.43) |  | 0.62 |  |  |  |
| Never-smokers |  |  |  |  |  |  |  |  |  |  |  |  |  |
| GPX1, rs3448==0 | 1.37 (1.13-1.65) |  | 25 | Ref (1.00) | 20 | 1.11 (0.57-2.17) | 38 | 1.85 (1.03-3.32) |  | 0.04 | 0.09 | 0.53 |  |
| GPX1, rs3448==1 | 1.01 (0.80-1.27) |  | 27 | Ref (1.00) | 27 | 0.98 (0.51-1.87) | 26 | 0.86 (0.44-1.66) |  | 0.64 |  |  |  |
| All |  |  |  |  |  |  |  |  |  |  |  |  |  |
| HSD3B1/B2, rs4659175==0 | 1.03 (0.87-1.21) |  | 44 | Ref (1.00) | 36 | 0.86 (0.51-1.44) | 39 | 0.90 (0.55-1.47) |  | 0.68 | 0.22 | 0.72 |  |
| HSD3B1/B2, rs4659175==1 | 1.12 (0.97-1.30) |  | 44 | Ref (1.00) | 28 | 0.66 (0.39-1.14) | 60 | 1.22 (0.76-1.97) |  | 0.34 |  |  |  |
| Never-smokers |  |  |  |  |  |  |  |  |  |  |  |  |  |
| HSD3B1/B2, rs4659175==0 | 0.97 (0.74-1.27) |  | 30 | Ref (1.00) | 26 | 0.87 (0.46-1.64) | 19 | 0.63 (0.33-1.19) |  | 0.16 | 0.04 | 0.39 |  |
| HSD3B1/B2, rs4659175==1 | 1.28 (1.07-1.52) |  | 22 | Ref (1.00) | 21 | 1.20 (0.60-2.138 | 45 | 2.15 (1.16-4.00) |  | 0.01 |  |  |  |
| All |  |  |  |  |  |  |  |  |  |  |  |  |  |
| HSD3B1/B2, rs10923823==0 | 0.93 (0.73-1.17) |  | 27 | Ref (1.00) | 24 | 1.03 (0.53-2.00) | 21 | 0.85 (0.44-1.63) |  | 0.62 | 0.06 | 0.59 |  |
| HSD3B1/B2, rs10923823==1 | 1.14 (1.01-1.29) |  | 61 | Ref (1.00) | 40 | 0.69 (0.44-1.09) | 79 | 1.25 (0.83-1.87) |  | 0.24 |  |  |  |
| Never-smokers |  |  |  |  |  |  |  |  |  |  |  |  |  |
| HSD3B1/B2, rs10923823==0 | 0.85 (0.61-1.19) |  | 18 | Ref (1.00) | 15 | 0.85 (0.37-1.94) | 10 | 0.50 (0.22-1.15) |  | 0.10 | 0.05 | 0.42 |  |
| HSD3B1/B2, rs10923823==1 | 1.24 (1.06-1.45) |  | 34 | Ref (1.00) | 32 | 1.13 (0.64-1.99) | 54 | 1.78 (1.05-3.01) |  | 0.03 |  |  |  |
| All |  |  |  |  |  |  |  |  |  |  |  |  |  |
| HSD3B1/B2, rs1047303==0 | 0.93 (0.77-1.12) |  | 48 | Ref (1.00) | 35 | 0.75 (0.46-1.23) | 38 | 0.80 (0.49-1.30) |  | 0.36 | 0.005 | 0.30 |  |
| HSD3B1/B2, rs1047303==1 | 1.22 (1.06-1.41) |  | 40 | Ref (1.00) | 29 | 0.78 (0.46-1.35) | 62 | 1.41 (0.88-2.28) |  | 0.12 |  |  |  |
| Never-smokers |  |  |  |  |  |  |  |  |  |  |  |  |  |
| HSD3B1/B2, rs1047303==0 | 0.94 (0.73-1.21) |  | 32 | Ref (1.00) | 25 | 0.77 (0.42-1.41) | 22 | 0.68 (0.36-1.29) |  | 0.24 | 0.007 | 0.39 |  |
| HSD3B1/B2, rs1047303==1 | 1.38 (1.12-1.69) |  | 20 | Ref (1.00) | 22 | 1.36 (0.67-2.69) | 42 | 2.03 (1.09-3.76) |  | 0.02 |  |  |  |
| All |  |  |  |  |  |  |  |  |  |  |  |  |  |
| HSD3B1/B2, rs7546652==0 | 0.93 (0.73-1.17) |  | 27 | Ref (1.00) | 24 | 1.03 (0.53-2.00) | 21 | 0.85 (0.44-1.63) |  | 0.62 | 0.05 | 0.59 |  |
| HSD3B1/B2, rs7546652==1 | 1.15 (1.02-1.30) |  | 60 | Ref (1.00) | 40 | 0.70 (0.45-1.11) | 79 | 1.28 (0.85-1.92) |  | 0.20 |  |  |  |
| Never-smokers |  |  |  |  |  |  |  |  |  |  |  |  |  |
| HSD3B1/B2, rs7546652==0 | 0.85 (0.61-1.19) |  | 18 | Ref (1.00) | 15 | 0.85 (0.37-1.94) | 10 | 0.50 (0.22-1.15) |  | 0.10 | 0.04 | 0.39 |  |
| HSD3B1/B2, rs7546652==1 | 1.25 (1.07-1.45) |  | 33 | Ref (1.00) | 32 | 1.17 (0.66-2.07) | 54 | 1.85 (1.09-3.13) |  | 0.02 |  |  |  |
| All |  |  |  |  |  |  |  |  |  |  |  |  |  |
| HSD3B1/B2, rs6428830==0 | 1.00 (0.84-1.19) |  | 48 | Ref (1.00) | 32 | 0.68 (0.41-1.12) | 42 | 0.95 (0.58-1.55) |  | 0.83 | 0.05 | 0.59 |  |
| HSD3B1/B2, rs6428830==1 | 1.17 (1.01-1.36) |  | 40 | Ref (1.00) | 32 | 0.89 (0.52-1.51) | 57 | 1.29 (0.80-2.07) |  | 0.26 |  |  |  |
| Never-smokers |  |  |  |  |  |  |  |  |  |  |  |  |  |
| HSD3B1/B2, rs6428830==0 | 1.04 (0.83-1.31) |  | 31 | Ref (1.00) | 23 | 0.79 (0.43-1.48) | 26 | 0.97 (0.52-1.83) |  | 0.92 | 0.04 | 0.39 |  |
| HSD3B1/B2, rs6428830==1 | 1.31 (1.06-1.63) |  | 21 | Ref (1.00) | 24 | 1.39 (0.70-2.77) | 37 | 1.72 (0.92-3.21) |  | 0.09 |  |  |  |
| All |  |  |  |  |  |  |  |  |  |  |  |  |  |
| NQO1, rs1800566==0 | 1.03 (0.89-1.19) |  | 63 | Ref (1.00) | 45 | 0.74 (0.48-1.15) | 61 | 0.90 (0.59-1.35) |  | 0.63 | 0.45 | 0.78 |  |
| NQO1, rs1800566==1 | 1.21 (1.02-1.44) |  | 25 | Ref (1.00) | 19 | 0.92 (0.46-1.82) | 39 | 1.84 (1.00-3.40) |  | 0.05 |  |  |  |
| Never-smokers |  |  |  |  |  |  |  |  |  |  |  |  |  |
| NQO1, rs1800566==0 | 1.05 (0.88-1.26) |  | 39 | Ref (1.00) | 35 | 1.08 (0.63-1.84) | 37 | 0.98 (0.58-1.66) |  | 0.94 | 0.03 | 0.39 |  |
| NQO1, rs1800566==1 | 1.49 (1.16-1.92) |  | 13 | Ref (1.00) | 12 | 0.99 (0.38-2.56) | 27 | 2.61 (1.13-6.00) |  | 0.02 |  |  |  |
| All |  |  |  |  |  |  |  |  |  |  |  |  |  |
| OGG1, rs1052133=0 | 1.19 (1.05-1.35) |  | 51 | Ref (1.00) | 33 | 0.70 (0.42-1.15) | 70 | 1.36 (0.89-2.07) |  | 0.12 | 0.03 | 0.59 |  |
| OGG1, rs1052133=1 | 0.88 (0.72-1.07) |  | 37 | Ref (1.00) | 31 | 0.82(0.47-1.42) | 30 | 0.70 (0.39-1.25) |  | 0.23 |  |  |  |
| Never-smokers |  |  |  |  |  |  |  |  |  |  |  |  |  |
| OGG1, rs1052133=0 | 1.27 (1.06-1.51) |  | 26 | Ref (1.00) | 27 | 1.31 (0.71-2.40) | 41 | 1.69 (0.98-2.94) |  | 0.06 | 0.31 | 0.65 |  |
| OGG1, rs1052133=1 | 0.98 (0.77-1.26) |  | 26 | Ref (1.00) | 20 | 0.72 (0.35-1.45) | 23 | 0.82 (0.41-1.65) |  | 0.60 |  |  |  |
| All |  |  |  |  |  |  |  |  |  |  |  |  |  |
| SRD5A1, rs824811==0 | 1.15 (1.00-1.32) |  | 46 | Ref (1.00) | 38 | 0.85 (0.52-1.38) | 62 | 1.27 (0.81-2.00) |  | 0.26 | 0.17 | 0.67 |  |
| SRD5A1, rs824811==1 | 0.98 (0.83-1.16) |  | 42 | Ref (1.00) | 26 | 0.69 (0.39-1.24) | 38 | 0.94 (0.55-1.59) |  | 0.82 |  |  |  |
| Never-smokers |  |  |  |  |  |  |  |  |  |  |  |  |  |
| SRD5A1, rs824811==0 | 1.34 (1.11-1.62) |  | 22 | Ref (1.00) | 26 | 1.24 (0.65-2.34) | 39 | 1.91 (1.04-3.50) |  | 0.04 | 0.02 | 0.39 |  |
| SRD5A1, rs824811==1 | 0.94 (0.77-1.15) |  | 30 | Ref (1.00) | 21 | 0.88 (0.44-1.74) | 25 | 0.83 (0.43-1.60) |  | 0.58 |  |  |  |
| All |  |  |  |  |  |  |  |  |  |  |  |  |  |
| XPC, rs2228000==0 | 1.00 (0.86-1.16) |  | 51 | Ref (1.00) | 33 | 0.77 (0.47-1.25) | 49 | 1.02 (0.64-1.62) |  | 0.94 | 0.07 | 0.59 |  |
| XPC, rs2228000==1 | 1.23 (1.03-1.47) |  | 37 | Ref (1.00) | 31 | 0.78 (0.45-1.34) | 51 | 1.25 (0.76-2.06) |  | 0.33 |  |  |  |
| Never-smokers |  |  |  |  |  |  |  |  |  |  |  |  |  |
| XPC, rs2228000==0 | 1.09 (0.90-1.31) |  | 25 | Ref (1.00) | 22 | 1.13 (0.58-2.17) | 28 | 1.27 (0.69-2.34) |  | 0.44 | 0.16 | 0.59 |  |
| XPC, rs2228000==1 | 1.30 (1.03-1.63) |  | 27 | Ref (1.00) | 25 | 0.91 (0.49-1.70) | 36 | 1.29 (0.71-2.33) |  | 0.38 |  |  |  |
| Clear differences in dose-response of acrylamide between genotypes | | | | | | | | | | | | | |
| All |  |  |  |  |  |  |  |  |  |  |  |  |  |
| CYP1B1, rs1056827==0 | 1.06 (0.90-1.26) |  | 42 | Ref (1.00) | 28 | 0.69 (0.41-1.18) | 51 | 1.01 (0.62-1.65) |  | 0.88 | 0.55 | 0.81 |  |
| CYP1B1, rs1056827==1 | 1.14 (0.97-1.33) |  | 46 | Ref (1.00) | 34 | 0.84 (0.50-1.40) | 48 | 1.26 (0.78-2.05) |  | 0.35 |  |  |  |
| Never-smokers |  |  |  |  |  |  |  |  |  |  |  |  |  |
| CYP1B1, rs1056827==0 | 1.04 (0.84-1.29) |  | 28 | Ref (1.00) | 21 | 0.75 (0.40-1.42) | 32 | 0.90 (0.49-1.66) |  | 0.80 | 0.15 |  |  |
| CYP1B1, rs1056827==1 | 1.38 (1.07-1.77) |  | 24 | Ref (1.00) | 24 | 1.19 (0.61-2.31) | 31 | 1.71 (0.91-3.22) |  | 0.09 |  |  |  |
| All |  |  |  |  |  |  |  |  |  |  |  |  |  |
| ESR2, rs2987983==0 | 1.18 (0.98-1.43) |  | 39 | Ref (1.00) | 24 | 0.67 (0.38-1.18) | 52 | 1.44 (0.86-2.43) |  | 0.15 | 0.29 | 0.76 |  |
| ESR2, rs2987983==1 | 1.03 (0.90-1.19) |  | 49 | Ref (1.00) | 40 | 0.89 (0.55-1.44) | 48 | 0.92 (0.59-1.45) |  | 0.73 |  |  |  |
| Never-smokers |  |  |  |  |  |  |  |  |  |  |  |  |  |
| ESR2, rs2987983==0 | 1.35 (1.08-1.69) |  | 22 | Ref (1.00) | 17 | 0.92 (0.46-1.85) | 38 | 1.84 (0.97-3.47) |  | 0.05 | 0.13 |  |  |
| ESR2, rs2987983==1 | 1.06 (0.86-1.30) |  | 30 | Ref (1.00) | 30 | 1.10 (0.60-2.00) | 26 | 0.92 (0.50-1.69) |  | 0.78 |  |  |  |
| All |  |  |  |  |  |  |  |  |  |  |  |  |  |
| MGC12965, rs1280350==0 | 1.07 (0.95-1.21) |  | 61 | Ref (1.00) | 43 | 0.78 (0.50-1.21) | 67 | 1.23 (0.82-1.86) |  | 0.31 | 0.77 | 0.90 |  |
| MGC12965, rs1280350==1 | 1.11 (0.86-1.43) |  | 27 | Ref (1.00) | 21 | 0.81 (0.43-1.55) | 33 | 0.86 (0.45-1.63) |  | 0.66 |  |  |  |
| Never-smokers |  |  |  |  |  |  |  |  |  |  |  |  |  |
| MGC12965, rs1280350==0 | 1.17 (1.01-1.36) |  | 35 | Ref (1.00) | 30 | 1.02 (0.59-1.78) | 47 | 1.75 (1.06-2.91) |  | 0.03 | 0.60 |  |  |
| MGC12965, rs1280350==1 | 1.06 (0.69-1.64) |  | 17 | Ref (1.00) | 17 | 0.95 (0.42-2.12) | 17 | 0.50 (0.20-1.22) |  | 0.11 |  |  |  |
| All |  |  |  |  |  |  |  |  |  |  |  |  |  |
| NOS2, rs944722==0 | 0.99 (0.78-1.26) |  | 34 | Ref (1.00) | 23 | 0.78 (0.42-1.44) | 29 | 0.75 (0.42-1.34) |  | 0.35 | 0.17 | 0.67 |  |
| NOS2, rs944722==1 | 1.13 (0.99-1.29) |  | 47 | Ref (1.00) | 40 | 0.82 (0.51-1.32) | 65 | 1.32 (0.84-2.06) |  | 0.19 |  |  |  |
| Never-smokers |  |  |  |  |  |  |  |  |  |  |  |  |  |
| NOS2, rs944722==0 | 1.09 (0.81-1.47) |  | 24 | Ref (1.00) | 17 | 0.92 (0.45-1.90) | 19 | 0.81 (0.41-1.60) |  | 0.55 | 0.36 |  |  |
| NOS2, rs944722==1 | 1.22 (1.03-1.44) |  | 23 | Ref (1.00) | 30 | 1.33 (0.71-2.46) | 42 | 1.87 (1.02-3.41) |  | 0.04 |  |  |  |
| All |  |  |  |  |  |  |  |  |  |  |  |  |  |
| PTGS2, rs5275==0 | 0.98 (0.83-1.16) |  | 47 | Ref (1.00) | 30 | 0.70 (0.41-1.19) | 41 | 0.92 (0.55-1.54) |  | 0.77 | 0.21 | 0.72 |  |
| PTGS2, rs5275==1 | 1.18 (1.02-1.37) |  | 41 | Ref (1.00) | 33 | 0.85 (0.50-1.45) | 59 | 1.30 (0.82-2.06) |  | 0.23 |  |  |  |
| Never-smokers |  |  |  |  |  |  |  |  |  |  |  |  |  |
| PTGS2, rs5275==0 | 1.03 (0.82-1.29) |  | 29 | Ref (1.00) | 22 | 0.83 (0.42-1.61) | 24 | 0.86 (0.43-1.72) |  | 0.68 | 0.26 |  |  |
| PTGS2, rs5275==1 | 1.27 (1.05-1.54) |  | 23 | Ref (1.00) | 25 | 1.41 (0.73-2.71) | 40 | 1.74 (0.97-3.13) |  | 0.06 |  |  |  |
| All |  |  |  |  |  |  |  |  |  |  |  |  |  |
| SRD5A1, rs8192120==0 | 1.00 (0.82-1.22) |  | 39 | Ref (1.00) | 25 | 0.76 (0.42-1.36) | 35 | 0.74 (0.43-1.27) |  | 0.28 | 0.44 | 0.78 |  |
| SRD5A1, rs8192120==1 | 1.13 (0.99-1.30) |  | 49 | Ref (1.00) | 39 | 0.81 (0.50-1.31) | 65 | 1.44 (0.92-2.25) |  | 0.10 |  |  |  |
| Never-smokers |  |  |  |  |  |  |  |  |  |  |  |  |  |
| SRD5A1, rs8192120==0 | 1.01 (0.78-1.30) |  | 24 | Ref (1.00) | 19 | 0.97 (0.47-1.97) | 24 | 0.79 (0.41-1.54) |  | 0.48 | 0.16 |  |  |
| SRD5A1, rs8192120==1 | 1.31 (1.09-1.59) |  | 28 | Ref (1.00) | 28 | 1.10 (0.60-2.02) | 40 | 1.81 (1.03-3.21) |  | 0.04 |  |  |  |

Hazard ratios are adjusted for age (years), age at menarche (years), age at menopause (years), parity (n children), ever use of oral contraceptives (yes/no), ever use of postmenopausal hormone treatment (yes/no), height (cm), body mass index (kg/m^2^), energy intake (kcal/day), and in the analyses for all women: smoking status (never/ex/current smoker), smoking quantity (n cigarettes/day), smoking duration (smoking years)
